# Impaired peptide amidation and risk of incident dementia in the Malmoe preventive project cohort

**DOI:** 10.1186/s13195-026-02173-7

**Published:** 2026-09-28

**Authors:** Paul Kaufmann, Yulia Ilina, Alice Giontella, Michaela Press, Katarina Nägga, Oskar Hansson, Harald Hampel, Andreas Bergmann, Olle Melander

**Affiliations:** 1 PAM Theragnostics GmbH, Neuendorfstr. 15a, 16761 Hennigsdorf, Germany; 2 Department of Clinical Sciences Malmö, Lund University, Malmö, Sweden; 3 Department of Health, Medicine, and Caring Sciences, Linköping University, Linköping, Sweden; 4 Clinical Department of Geriatrics and Palliative Medicine, Region Östergötland, Linköping, Sweden; 5 Clinical Memory Research Unit, Department of Clinical Sciences Malmö, Faculty of Medicine, Lund University, Malmö, Sweden; 6 Alzheimer Precision Medicine (APM), Sorbonne University, AP-HP, Pitié-Salpêtrière Hospital, Boulevard de L’hôpital, Paris, France; 7 Department of Emergency and Internal Medicine, Skåne University Hospital, Malmö, Sweden

**Keywords:** Alzheimer’s disease, Peptidylglycine α-amidating monooxygenase (PAM), Peptide amidation, Adrenomedullin, Neurovascular dysfunction, Blood-based biomarker, Population-based study

## Abstract

**Background:**

Peptidylglycine α-amidating monooxygenase (PAM) is the key enzyme responsible for the C-terminal amidation of multiple vasoactive and neuroactive peptide hormones. Among these, amidated bioactive adrenomedullin (bio-ADM) is a critical regulator of endothelial- and blood–brain barrier (BBB) integrity and vascular homeostasis. Impaired peptide amidation may contribute to endothelial dysfunction and neurovascular dysregulation, mechanisms implicated in the pathogenesis of Alzheimer’s disease (AD).

**Methods:**

In 4543 dementia-free participants from the Malmö preventive project (MPP), plasma levels of mid-regional pro-adrenomedullin (MR-proADM), bio-ADM, and PAM activity (PAM-AMA) were measured at baseline, and incident dementia was ascertained through national registry data during follow-up. Associations were evaluated using Cox proportional hazards models with multivariable adjustment for common risk factors, false discovery rate correction, BMI-stratified analyses, and sensitivity analyses.

**Results:**

During a mean follow-up of 4.75 ± 1.3 years, 205 participants developed incident AD and 93 developed vascular dementia (VD). Lower PAM-AMA was associated with increased risk of incident AD (adjusted hazard ratio per 1 standard deviation decrease (aHR): 2.30; 95% confidence interval (CI): 1.31–4.02; q = 0.01). Lower bio-ADM levels were also associated with increased Alzheimer’s risk (aHR: 1.62; 95% CI: 1.13–2.33; q = 0.01). A reduced bio-ADM/MR-proADM ratio and its interaction with PAM-AMA further strengthened AD risk estimates. Associations were independent of APOE ε4 carrier status, more pronounced among individuals with BMI < 30 and remained directionally consistent in sensitivity analyses. No consistent associations were observed for VD after multivariable adjustment.

**Conclusions:**

Reduced PAM-AMA and lower bio-ADM levels were associated with increased risk of incident AD, but not VD. A reduced ADM-ratio further indicates precursor peptide accumulation. These findings suggest that impaired peptide hormone amidation may represent an early systemic marker of neurovascular vulnerability and support further investigation of C-terminal peptide amidation pathways in translational AD research, including studies with a longer follow-up period and biomarker-confirmed dementia diagnoses.

**Supplementary Information:**

The online version contains supplementary material available at https://doi.org/10.1186/s13195-026-02173-7.

## Background

Dementia is a disorder of the central nervous system associated with a progressive cognitive impairment which encompasses various subtypes such as Alzheimer’s disease dementia (AD) and vascular dementia (VD). AD is the most prevalent type accounting for 60–70% of all dementia cases, followed by VD constituting approximately 10–20% [1]. As of 2021, over 57 million people globally are affected by dementia while its global prevalence is projected to exceed 140 million cases by 2050 [1, 2]. Growing evidence suggests vascular impairment as an early and critical event in pathogenesis of all major dementia forms [3, 4]. Vascular and cerebrovascular dysfunction, including blood–brain barrier (BBB) disruption, cerebral hypoperfusion, and microvascular lesions, have been implicated as early events in several dementia types, such as AD and VD [5]. They precede the accumulation of Aβ plaques and tau tangles, suggesting a pivotal role in disease initiation [5–7]. BBB integrity, controlled among others by several amidated peptide hormones, is essential for clearing neurotoxic proteins and maintaining cerebral homeostasis. It is compromised in AD, exacerbating amyloidosis, tauopathy, and neuroinflammation [8, 9].

A critical enzymatic mechanism that sustains vascular and neuronal peptide signalling is C-terminal amidation, catalysed by the enzyme peptidylglycine α-amidating monooxygenase (PAM) [10]. Numerous peptide hormones require the C-terminal amidation as their final biosynthesis-step, a posttranslational modification essential for receptor binding and biological activity [11]. Such peptides are typically referred to as “amidated peptide hormones”, including neuropeptide Y (NPY) [12], substance P [13], vasoactive intestinal peptide (VIP) [14], pituitary adenylate cyclase-activating polypeptide (PACAP) [15, 16], calcitonin gene-related peptide (CGRP) [17], α-melanocyte-stimulating hormone (α-MSH) [18], and adrenomedullin (ADM) [19]. PAM circulates in plasma and its amidating activity (PAM-AMA) can be measured from human bodily fluids, including serum and plasma [11, 20–22]. Early studies reported reduced PAM-AMA in cerebrospinal fluid (CSF) and brain tissues of patients with prevalent AD [23] and a recent study by Imam et al. analysed large-scale proteomic data from multiple cohorts, revealing a consistent reduction of PAM in AD and other neurodegenerative diseases [24].

Because PAM catalyses the amidation of numerous neuroprotective and vasoactive peptides, impaired PAM functionality may underlie systemic deficits in vascular regulation. Amidated peptides were reported to protect the endothelium (bio-ADM, VIP), enhance cerebral perfusion (PACAP, CGRP), stabilize the BBB (bio-ADM, CGRP) and reduce amyloid-ß depositions (PACAP, VIP, α-MSH, NPY) [12, 14–19]. In AD, reduced levels of several amidated peptides, including PACAP, Substance P and NPY, have been reported, suggesting a common deficit in neuropeptide signalling [12, 13, 25]. As PAM represents the shared, rate-limiting biosynthetic step for all peptides requiring C-terminal amidation, a reduction in PAM activity may constitute a common mechanism underlying what have previously been described as distinct, peptide-specific deficits in dementia, suggesting that PAM-AMA may serve as a measurable proxy for the cumulative impairment of multiple neuroprotective and vasoactive peptide-hormones.

**Fig. 1:**
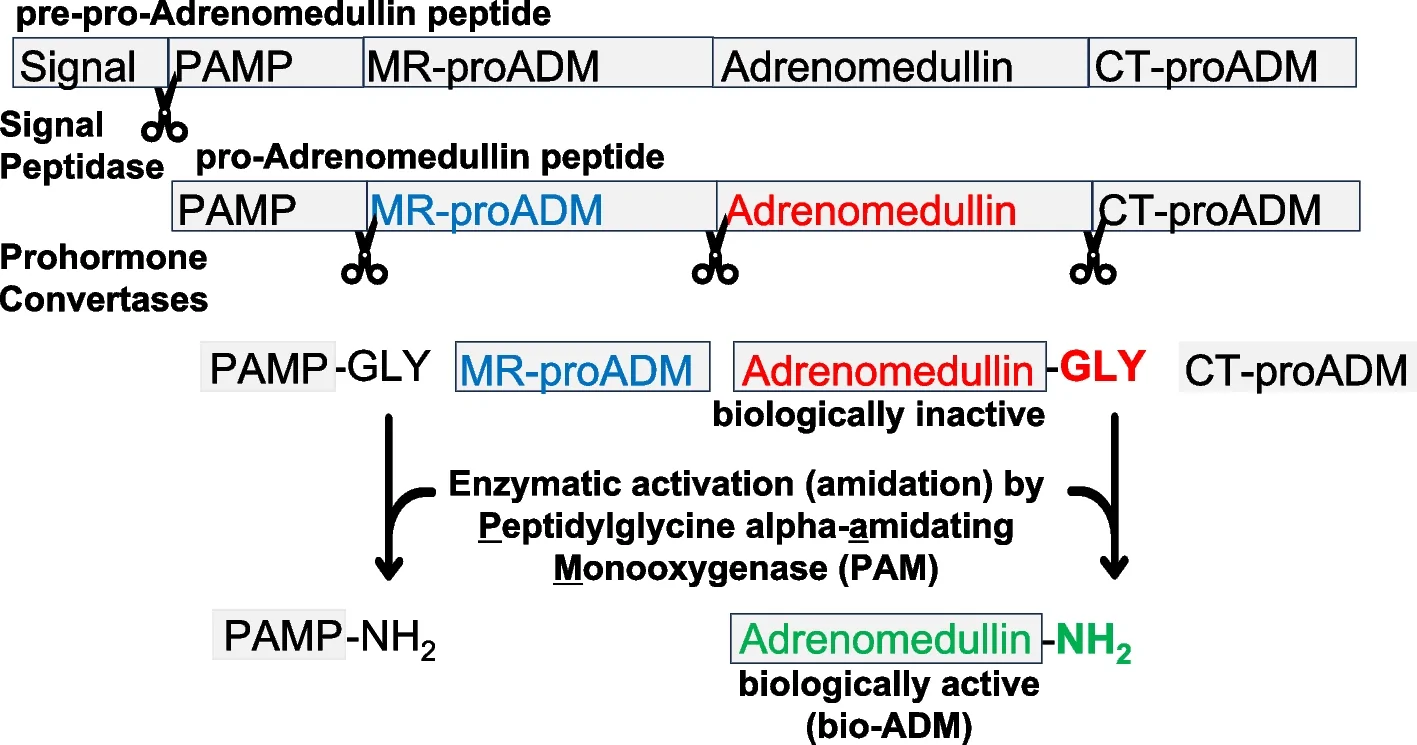
Schematic representation of the processing of pre-pro-adrenomedullin. After proteolytic processing by several prohormone convertases, the peptides PAMP-Gly (Proadrenomedullin N-terminal 20 peptide with a C-terminal Glycine residue), MR-proADM, ADM-Gly (Adrenomedullin with a C-terminal Glycine residue) and CT-proADM (C-terminal pro-Adrenomedullin) emerge. Biologically inactive PAMP-Gly and ADM-Gly are further amidated by the enzyme PAM to result in biologically active PAMP and bio-ADM, both carrying a C-terminal amide

The biologically active, amidated ADM (bio-ADM), plays a crucial role in vascular regulation and integrity, in maintaining vascular tone, and it is a potent endothelial maintenance hormone with strong vasodilatory actions [26]. It is further considered as a key endogenous regulator of cerebral blood flow and BBB integrity, thereby contributing to the homeostasis of the brain and to neuroprotection [27–30]. ADM is synthesized as a large precursor peptide, which is processed into several circulating fragments, including mid-regional pro-adrenomedullin (MR-proADM) and the inactive ADM-Gly (Fig. 1) [27]. ADM-Gly serves as a substrate to PAM and is the precursor of bio-ADM [27]. MR-proADM is a stable, biologically inactive fragment of pro-ADM that serves as a reliable surrogate marker for ADM precursor levels and has been studied as a biomarker in neurovascular and neurodegenerative disorders. Its elevated plasma levels were associated with an elevated risk of MCI to AD progression [31, 32]. In a proteomics-based approach, elevated plasma levels of MR-proADM were associated with an increased dementia risk [33]. Although MR-proADM has served as a convenient, stable surrogate for circulating ADM levels, it is bio-ADM that exerts the endothelial-stabilizing and neuroprotective effects. Elevated plasma levels of bio-ADM were associated with several conditions and diseases, particularly in vascular and ischemic contexts, where it exerts endothelial-stabilizing and neuroprotective effects [34–36]. However, data on bio-ADM as a plasmatic biomarker in dementias were not reported to date. ADM signalling pathways were proposed to play a protective neurovascular role in AD by maintaining endothelial integrity, promoting angiogenesis and reducing neuroinflammation [37]. Via the CLR/RAMP2 receptor complex, bio-ADM reduces endothelial permeability and preserves BBB integrity through pathways involved in nitric oxide production and tight-junction maintenance [29, 30, 38, 39]. Further, these mechanisms contribute to suppression of endothelial adhesion molecule expression and pro-inflammatory cytokine signalling which makes them relevant to the early neurovascular changes and ADM pathway dysregulation observed in AD pathology [36–39].

The objective of this study was to investigate the relationship between plasma levels of PAM-AMA with the risk of developing dementia, in particular AD and VD, alongside with levels of bio-ADM and MR-proADM. Using a sub-cohort of 4543 individuals from the Malmö Preventive Project (MPP), this study investigated whether levels of PAM-AMA and its product bio-ADM are changed in incident dementia and whether these biomarkers could predict incident dementia.

## Materials and methods

### MPP study population

The Malmö Preventive Project (MPP), a Swedish single-centre, prospective, population- based study is described elsewhere [40, 41]. In short: the study enrolled 33,346 individuals living in Malmö to explore CV risk factors in the general population. Between 2002 and 2006 18,240 of the surviving original participants underwent re-examination (MPP-RES, 70.5% participation rate). For this study, the used subcohort of plasma samples comprised a computer-generated random sample of *n* = 4620 MPP re-examination participants who had not previously participated in the Malmö Diet and Cancer (MDC) study, supplemented by all prevalent and incident dementia cases identified within the full MPP-RES cohort. Consequently, the remaining MPP-RES participants contained, by design, no dementia cases. The re-examination visit was considered the baseline time point throughout the present analyses. Within the selected study population, 62 participants were diagnosed with prevalent dementia before re-examination and were excluded from the analysis, 15 additional subjects were excluded due to missing baseline parameters, resulting in a study sample of *n* = 4543 subjects. Baseline characteristics of the resulting sample and the remaining MPP-RES population are compared in supplementary table S1A. Prevalent dementia was defined as a dementia diagnosis recorded prior to the re-examination visit, whereas incident dementia was defined as a first dementia diagnosis occurring after baseline during follow-up. The first incident dementia diagnosis occurred 51 days after the re-examination visit, supporting the definition of the re-examination as baseline and the classification of all subsequent dementia diagnoses as incident events. Before entering MPP-RES, all participants signed a written informed consent form. The study was approved by The Regional Ethical Review Board at Lund University, Sweden (LU 244–02) and complied with the Declaration of Helsinki.

#### Dementia diagnosis

Dementia diagnosis was obtained from the Swedish National Patient Register (SNPR) covering the period from the start of the MPP study until December 31, 2009. The diagnosis was recorded using different versions of the International Classification of Diseases (ICD) codes: 290 and 293 (ICD-8), 290 and 331 (ICD-9), and F00, F01, F03, and G30 (ICD-10). The SNPR has included all in-patient care in Sweden since 1987. From 2000 onwards, it also started recording outpatient visits, including day surgeries and psychiatric care. Alzheimer’s disease and vascular dementia were diagnosed using DSM-IV (Diagnostic and Statistical Manual of Mental Disorders, Fourth Edition) criteria. DSM-IV defines dementia as an acquired and persistent decline in cognitive function characterised by memory impairment accompanied by at least one additional cognitive deficit, including aphasia, apraxia, agnosia, or impaired executive functioning. These deficits must represent a decline from a previous level of functioning, result in significant impairment in social or occupational activities, and not occur exclusively during episodes of delirium [42]. Diagnosis was based on clinical assessment and functional impairment and did not incorporate biological or pathophysiological biomarkers. Information on detailed neuropsychological testing protocols and standardized neuroimaging criteria was not uniformly available across cases; diagnosis therefore relied on clinical assessment and available routine diagnostic data as recorded in medical records. A research physician validated diagnosis by reviewing medical records and available neuroimaging data, consulting a geriatrician specializing in cognitive disorders for complex cases, resulting in a total of 471 identified individuals with a validated dementia diagnosis in the SNPR. Given the reliance on clinical diagnostic criteria, individuals classified as having Alzheimer’s disease may have had mixed dementia pathology, which cannot be reliably distinguished in registry-based data and should be considered a limitation of the study. In the study sample of *n* = 4543 analysed in this work, 205 individuals had an incident AD diagnosis and 93 had an incident VD diagnosis. Of these, 14 individuals received both an AD and a VD diagnosis, reflecting a mixed dementia phenotype. These participants were not excluded from the subtype-specific analyses. Instead, each dementia endpoint was evaluated independently: individuals were classified as incident AD cases regardless of additional VD diagnosis, and analogously, as incident VD cases irrespective of concurrent AD diagnosis. The analysed dataset considers follow-up times and diagnosis made from the date of rescreening through 31.12.2009.

Prevalent diagnosis of cardiovascular diseases and diabetes mellitus was obtained from the Swedish National Patient Register (SNPR) for the period from the start of the MPP study until December 31, 2009. Cardiovascular disease events comprise cumulative prevalent cases of stroke, atrial fibrillation, heart failure, aortic stenosis and major adverse cardiac events, including coronary events, coronary artery bypass grafting and percutaneous coronary intervention. CV disease events were combined with prevalent diabetes events as a dichotomous cardiometabolic variable.

#### Biomarker measurement

In the study sample of *n* = 4543, circulating levels of mid-regional pro-adrenomedullin (MR-proADM) were determined using the Thermo Scientific BRAHMS MR-proADM KRYPTOR assay [43] and were previously reported by Holm et al., 2017 [44]. Levels of PAM amidating activity (PAM-AMA) were determined from serum samples as previously described [45] using a 2-step in vitro activity assay that utilises synthetic human 1–53 ADM-Gly as a substrate. Briefly, in the first step the PAM-catalysed amidation of ADM-Gly is performed under optimized cofactor conditions (copper and ascorbate). In the second step, the resulting 1–52 amidated ADM (bio-ADM) as the product of PAM catalysed amidation was quantified and used to calculate PAM-AMA in AMA-Units [45]. 1 AMA-Unit is defined as 1 µg bio-ADM generated in 1 L of sample in 1 h. The quantification of bio-ADM was performed using a luminescent sandwich immunoassay, which was previously described [46]. The same assay was used for determination of circulating levels of bio-ADM from EDTA-plasma. Therefore, the main biomarkers analysed included MR-proADM in nM, bio-ADM in pg/mL, PAM-AMA in AMA-Units [45]. Additionally, the molar ratio of bio-ADM/MR-proADM (ADM-ratio) was investigated, wherein a molecular weight of 6028.9 Da was considered for bio-ADM in the transformation for mass- to molar concentrations. An interaction term, calculated as PAM-AMA in AMA-Units x ADM-ratio, was also included in the analyses. This interaction term was included to capture the combined effect of enzymatic amidation activity (PAM-AMA) and the relative conversion of precursor to active hormone (ADM-ratio), reflecting functional PAM-dependent bio-ADM availability. The distribution of all primary biomarkers, the ADM-ratio and the interaction term was right-skewed as per analysis using the Shapiro–Wilk test, therefore log-transformation was utilised to normalize their distributions and facilitate interpretability.

#### APOE-genotyping

Genome sequencing was performed by the Genomic core facility at Erasmus University Medical Center (Rotterdam) using Illumina chip genotyping (Illumina Infinium Global Screening Array "GSAMD-25v1-0_20011747_A1"). The resulting genotype data were used for downstream quality control, imputation, and variant analyses. Quality control steps were performed using plink tool (plinkv2.0) [47]: high-LD genomic regions, individuals with more than 0.1% missingness rate (–mind 0.1) or SNPs with a missingness higher than 0.1% (–geno 0.1) or with a Hardy Weinberg equilibrium below 1e-15 (–hwe) were filtered out. Analysis was restricted to European and not related individuals having consistency between estimated genetic sex and reported sex and not affected by sex chromosome aneuploidy. Relatedness was assessed using identity-by-descent estimates, and one individual from each related pair exceeding predefined relatedness thresholds was excluded. Genetic ancestry was inferred using principal component analysis in comparison with reference populations, and only individuals clustering with European ancestry were included. Imputation was done at Michigan Imputation server (https://imputationserver.sph.umich.edu/) using *hrc.r.1.1.2016* as reference panel. APOE genotypes were derived from the two defining single-nucleotide polymorphisms rs429358 and rs7412, which were obtained from imputed genotype data following standard post-imputation quality control.

For each individual, the combination of the two APOE alleles results in one of six possible genotypes: ε2/ε2, ε2/ε3, ε2/ε4, ε3/ε3, ε3/ε4, or ε4/ε4. These alleles correspond to the three major APOE protein isoforms—APOE ε2, APOE ε3, and APOE ε4—which differ by amino acid substitutions at positions 112 and 158. The isoforms are determined by the nucleotide combinations at rs429358 and rs7412 that define the ε2 (T–T), ε3 (T–C), and ε4 (C–C) alleles. Individuals carrying at least one ε4 allele (ε2/ε4, ε3/ε4, or ε4/ε4) were classified as APOE ε4 carriers, whereas those with ε2/ε2, ε2/ε3, or ε3/ε3 genotypes were considered non-carriers [48]. In the analysed study sample of *n* = 4543 participants, APOE status was available for *n* = 4388 individuals.

### Statistical analyses

#### Descriptive statistics

Between dementia positive and individuals free of dementia, group differences in continuous variables were tested with Mann–Whitney U test or ordinary ANOVA and categorical variables were compared with Pearson’s Chi-square test. Distributions of biomarkers were assessed using histograms and density plots. Skewness was calculated for each biomarker and categorised as symmetric, moderately skewed, or highly skewed using the Shapiro–Wilk test. Biomarkers were categorised into quartiles for Cox regression analyses, with the highest quartile (Q4) set as the reference.

#### Continuous biomarker analyses

Associations between biomarkers and dementia outcomes were analysed using Cox proportional hazards models with time-to-event as the underlying time scale. For each endpoint, the biomarkers were modelled as continuous predictors. The biomarkers underwent inverse log-transformation to facilitate interpretation of lower biomarker levels as higher risk. Cox models were parameterized to estimate hazard ratios (HRs) per 1 standard-deviation (SD) increase in the inverse log-transformed biomarker, corresponding to lower biomarker levels on the original scale. For each biomarker and endpoint, three model specifications were fitted: a univariable model, a multivariable model adjusted for age, sex, systolic blood pressure (SBP), antihypertensive treatment (AHT), current smoking status, high-density lipoprotein (HDL), history of prior cardiometabolic disease (CMD) and a multivariable model that additionally included BMI as covariable. Additionally, the univariable and multivariable models were repeated in the total cohort after stratification for BMI (BMI < 30 kg/m^2^ and BMI ≥ 30 kg/m^2^) using the same model specifications and covariates as in the main analyses. To account for multiple testing across biomarkers, false discovery rate (FDR) control was applied using the Benjamini–Hochberg method. Bio-ADM, MR-proADM, and PAM-AMA were defined as co-primary biomarkers in statistical analyses. FDR correction was applied to p-values derived from continuous Cox regression analyses for these biomarkers, separately for each endpoint and model specification. Resulting adjusted q-values < 0.05 were considered statistically significant. The ADM-ratio and IT were analysed as secondary, derived measures and were therefore not included in the FDR correction.

#### APOE ε4 adjusted continuous analyses

Additional analyses restricted to participants with known APOE genotype *(n* = 4388) were performed. In this subset, a binary covariate (APOE4_pos) indicating the presence of at least one APOE ε4 allele (heterozygous or homozygous carrier) was created. The same continuous biomarker specifications as in the main analyses were applied including “APOE4_pos” as covariate. Cox models were fitted for each biomarker and endpoint under three scenarios as described in the “continuous biomarker analyses” section. The formal evaluation of the influence of APOE ε4 on biomarker effects was conducted as a sensitivity analysis comparing multivariable models with and without consideration of the APOE status as covariate for each biomarker and endpoint.

#### Categorical biomarker analyses

The dose–response relationships between biomarker levels and dementia risk were evaluated after stratification of all log-transformed biomarkers as quartiles (Q1-Q4). Quartile cut points were derived on the log scale. The highest quartile (Q4) was used as the reference category in all models. Cox proportional hazards models were fitted separately for incident AD and incident VD, with follow-up time defined as in the main analyses. Three model specifications were used: an unadjusted model, a multivariable model adjusted for age, sex, SBP, AHT, current smoking, HDL, and history of CMD, and a multivariable model additionally adjusted for BMI. To formally assess dose–response trends across biomarker quartiles, an ordinal quartile variable was constructed, and the Cox models were refitted including this variable as a linear term, using the same covariate sets as in the unadjusted, “adjusted”, and “adjusted + BMI” scenarios. The p-value for the quartile term from these models was interpreted as a test for linear trend across increasing biomarker levels. All categorical analyses were conducted in the full cohort and then repeated in BMI-stratified subgroups (BMI < 30 kg/m^2^ and BMI ≥ 30 kg/m^2^). In the subgroup analyses, unadjusted and adjusted models were fitted as described above, with identical covariate sets apart from the subgroup restriction.

For each Cox model, overall model fit was evaluated using the Likelihood-Ratio (LR) Chi-square statistic and corresponding *p*-value obtained from the model’s log-likelihood comparison.

For each biomarker, Kaplan–Meier plots were generated to visualize time-to-event distributions across quartiles. The time-to-event (follow-up time) was calculated as the difference between the screening date and the date of diagnosis.

#### Sensitivity analyses

To evaluate whether early, subclinical dementia cases biased baseline biomarker levels, prespecified reverse-causation sensitivity analyses using a lagged Cox model were performed. For incident AD and VD the continuous Cox proportional hazards analyses were recreated after recoding all events in the first two years of follow-up as censored. Participants who developed the respective dementia outcome within ≤ 2 lag years after baseline were retained in the risk set but their incident AD or VD event indicator was set to 0, while all other follow-up times and covariates remained unchanged. Thus, the time-scale and censoring structure of the original models were preserved. These reverse-causation analyses were conducted for all primary biomarkers, the ADM-ratio and the interaction term using the same model specifications and adjustments as in the primary analyses. Hazard ratios were estimated per 1-SD increase in the inverse log-transformed biomarker values, corresponding to lower biomarker levels on the original scale, consistent with the main models. All reverse-causation analyses were first performed in the total cohort and then repeated in BMI-stratified subgroups (BMI < 30 and BMI ≥ 30 kg/m^2^), using identical lag definitions and covariate sets. The same two-year lag definition was applied to categorical analyses based on biomarker quartiles. Quartile-based Cox proportional hazard models were re-estimated after censoring the events occurring within the first two years of follow-up, applying identical covariate adjustments and reference categories as in the primary analyses.

To assess the robustness of AD-specific associations, additional sensitivity analyses were performed restricting the outcome definition to incident AD without VD cases. Individuals who developed VD during follow-up were excluded from these analyses. Continuous Cox proportional hazards models were re-estimated using identical biomarker specifications, covariate sets, and model adjustments as in the primary continuous analyses.

#### Internal validation of model discrimination

To assess internal model stability and potential overfitting of the continuous AD models, bootstrap-based analysis was performed using the concordance index (C-index). Base models were defined as multivariable adjusted Cox models including dementia risk factors as described in "Continuous biomarker analyses" section, without biomarker terms. Base models were compared with biomarker-extended models using identical covariable sets. For each base and biomarker-extended model, optimism was estimated by comparing model discrimination in bootstrap samples with performance in the original dataset, and optimism-corrected C-indices were obtained by subtracting the estimated optimism from the apparent C-index. Optimism-corrected C-indices were used to evaluate the robustness of model discrimination.

#### Statistical software

Biomarker distributions and Cox proportional hazard regression were performed using R (version 4.4.2), with packages including *survival*, *dplyr*, and *survminer*. C-indices for multivariable Cox models were obtained using bootstrap-based concordance estimation implemented in the *survcomp* package. Testing of group differences and visualization of Kaplan–Meier plots and Box-plots were performed using GraphPad Prism (version 10.4.0 (621)).

### Large language model use

ChatGPT (OpenAI) was used to assist in generating and refining selected R code snippets and to improve clarity and grammar in parts of the manuscript. Additionally, the Nature Research Assistant tool (https://natureresearchassistant.com/) was consulted to support manuscript revision and structural refinement.

## Results

A total of *n* = 4543 individuals with available measurements of the plasma activity of PAM-AMA, MR-proADM and bio-ADM levels and without a history of dementia prevalence were included in the analysis. Baseline characteristics of the selected cohort, the selected cohort after exclusion of incident dementia, and the remaining MPP-RES population are summarized in supplementary table S1A. Overall, the cohorts were broadly comparable across most baseline variables. The most notable difference was a higher proportion of men in the selected cohort (*n* = 4543), consistent with exclusion of participants who had also participated in the MDC study, which included a higher proportion of women [49]. Modest differences in vascular comorbidity reflected the dementia-enriched sampling design.

Participants were followed from the re-examination visit until dementia diagnosis, censoring or end of registry follow-up, with a mean follow-up time (± standard deviation (SD)) of 4.75 ± 1.3 years. During this period, 284 individuals developed incident dementia, defined here as the combined incidence of AD and VD (incident ADVD). Study participants who developed incident dementia were older, more often female, less likely to be current smokers and more frequently had a history of cardiovascular or cardiometabolic disease (Table 1). Subjects with incident dementia had a lower body mass index (BMI), a lower diastolic blood pressure (DBP), lower blood glucose levels, lower triglyceride levels and higher plasmatic high-density lipoprotein (HDL) concentrations (Table 1). No significant differences were observed for systolic blood pressure (SBP), antihypertensive medication (AHT) use, plasma low-density lipoprotein (LDL) plasma cholesterol. Of the total cohort, APOE genotype data were available for *n* = 4388 individuals (96.6%), wherein *n* = 1327 (29.2%) were APOE ε4 carriers. Among individuals with incident dementia, 12.3% were homozygous and 44.7% were heterozygous APOE ε4 carriers. In contrast, 2.1% and 25.3% of dementia-free individuals were homozygous and heterozygous APOE ε4 carriers, respectively.

**Table 1:** Baseline characteristics of the analysed MPP study population (*n* = 4543) by dementia positive and negative subjects

Characteristics	Incident dementia positive (*n* = 284)	Free of dementia (*n* = 4259)	*p*-Value
Age (years)	73.9 ± 4.4	69.5 ± 6.2	< 0.0001
Sex, (% male)	58.1	71.0	< 0.0001
Current smoking, (%)	13.0	18.8	0.02
Systolic BP (mmHg)	143.7 ± 20.1	145.7 ± 20.3	n.s
Diastolic BP (mmHg)	81.8 ± 10.4	83.8 ± 10.8	0.005
Antihypertensive treatment, (%)	44.7	38.9	n.s
Plasma LDL (mM)	3.6 ± 1	3.6 ± 1	n.s
Plasma HDL (mM)	1.45 ± 0.4	1.37 ± 0.4	0.001
Plasma cholesterol (mM)	5.6 ± 1.2	5.6 ± 1.1	n.s
Glucose (mM)	5.7 ± 1.2	5.9 ± 1.5	0.03
BMI	25.6 ± 3.7	27.2 ± 4.2	< 0.0001
Plasma triglycerides (mM)	1.16 ± 0.58	1.24 ± 0.63	0.02
History of CMD (%)	33.5	27.6	0.03
History of CVD (%)	26.1	16.8	< 0.0001
Stroke prevalence (%)	8.8	4.0	< 0.0001
APOE ε4 homozygous (%)	12.3	2.1	< 0.0001
APOE ε4 heterozygous (%)	44.7	25.3	< 0.0001
APOE ε4 negative (%)	40.5	69.1	< 0.0001
APOE status missing (%)	2.5	3.5	n.s
PAM-AMA (AMA-Units)	12.6 ± 3.3	12.9 ± 3.3	n.s
bio-ADM (pg/mL)	15.8 ± 10.1	16.4 ± 12.1	0.01
MR-proADM (nM)	0.79 ± 0.2	0.75 ± 0.2	0.0002
ADM-ratio (× 10–^3^)	3.3 ± 1. 8	3.7 ± 2.7	< 0.0001

The APOE status was missing in *n* = 155 of 4543 specimen. Data is presented as mean ± standard deviation (SD) or as percentage of the respective subgroup. Differences in continuous variables were tested with ordinary ANOVA, categorical variables were compared with Pearson’s Chi-square test

*BP* Blood pressure, *LDL* Low-density lipoprotein, *HDL* High-density lipoprotein, *CMD* Cardiometabolic disease, *CVD* Cardiovascular disease, *bio-ADM* Bioactive Adrenomedullin, *MR-proADM* Mid-regional pro-Adrenomedullin, *ADM-ratio* Molar ratio of bio-ADM/MR-proADM, *PAM-AMA* Peptidylglycine-alpha amidating monooxygenase amidating activity

**Fig. 2:**
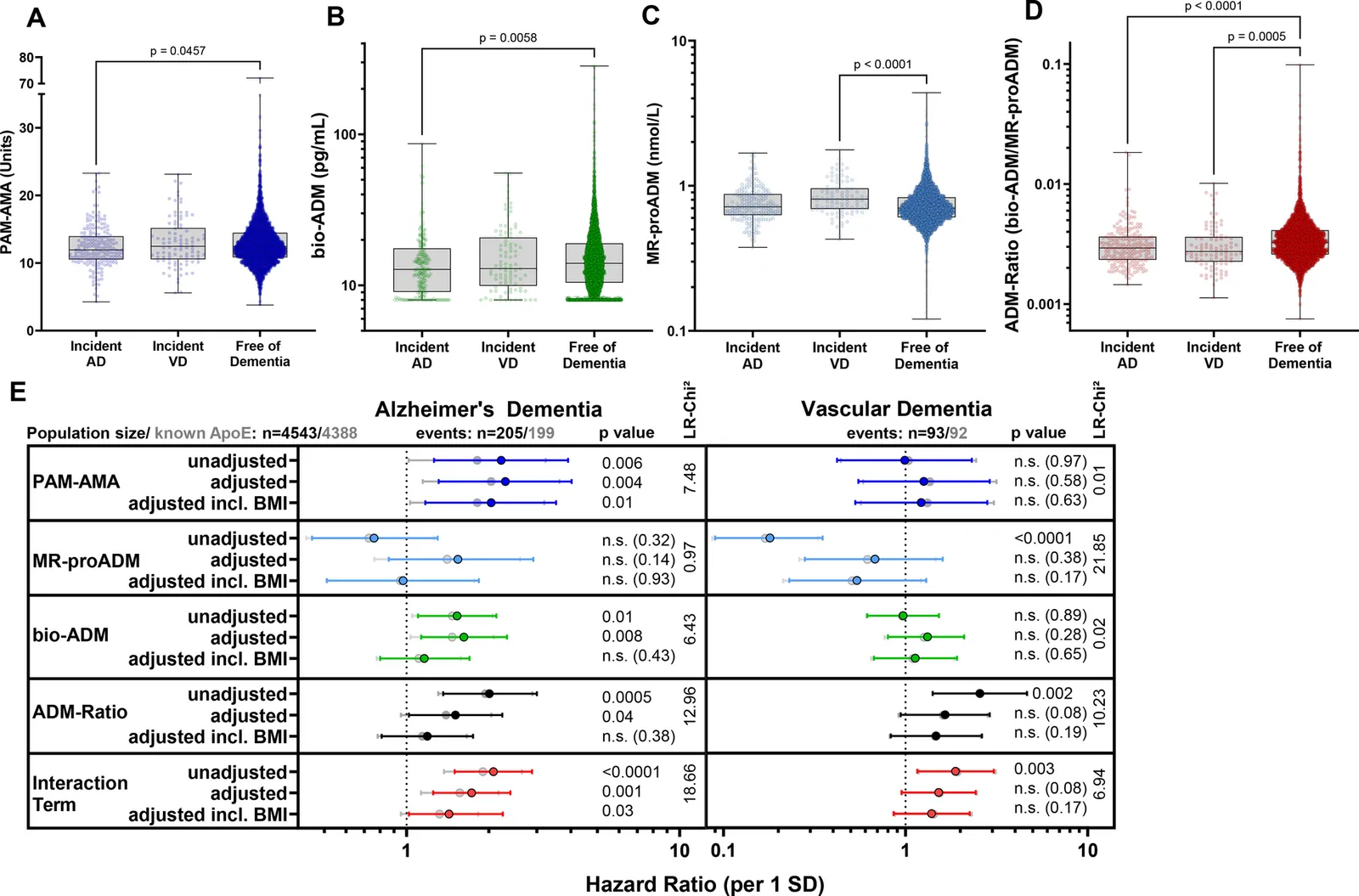
Boxplot (**A**-**D**) visualisation of PAM-AMA levels (**A**), bio-ADM levels (**B**), MR-proADM levels (**C**) and ADM-ratio (**D**) across dementia subtypes in comparison to dementia free individuals. Significance of biomarker differences in dementia subtypes compared to dementia free individuals was tested with Kruskal–Wallis test for multiple comparisons. The p-values are shown for significant differences within each panel. Forest plot (**E**) of Hazard Ratios per 1 SD for the biomarkers PAM-AMA, MR-proADM, bio-ADM, the ADM-ratio and the Interaction Term. The HRs are shown with 95% confidence intervals (CI). The HRs are presented either in an unadjusted form (unadjusted) or after adjustment for age, sex, SBP, AHT, smoking, HDL, and prior history of cardiometabolic disease (adjusted). An additional analysis including BMI as a covariate is shown as “adjusted incl. BMI”. Each analysis was performed for the indications incident AD and VD. Additionally, the presence of APOE ε4 was considered in all scenarios (unadjusted, adjusted and adjusted incl. BMI). Within the analysed study population the APOE status was present for *n* = 4388 subjects, the event numbers in the study population with known APOE status are given in light grey. APOE ε4 status was included as an additional covariate in the adjusted analyses for each biomarker and endpoint. The calculated HRs incl. 95% CI are shown in light grey for comparison. Numerical values for the full cohort are summarized in Table 2. Numerical values for the cohort with known APOE status are summarized in supplementary table S2, including ΔaHR values for adjustments with and without APOE ε4 presence as a covariable. All HRs are expressed per 1-SD decrease in the respective biomarker following inverse log-transformation, such that HR > 1 indicates higher risk associated with lower biomarker levels

Baseline biomarker levels differed between groups: individuals with incident dementia had significantly lower plasma levels of bio-ADM (15.8 pg/mL) and significantly higher plasma levels of MR-proADM (0.79 nM) when compared to dementia-free subjects (16.4 pg/mL and 0.75 nM, respectively, Table 1). The molar ratio of bio-ADM/MR-proADM (ADM-ratio) was significantly lower in subjects with incident dementia (0.033 vs 0.037, respectively, Table 1). PAM-AMA was also reduced in individuals with incident dementia, but this difference was not statistically significant (12.6 AMA-Units vs 12.9 AMA-Units, Table 1). Further stratification by dementia subtypes (incident AD and VD, supplementary table S1B) revealed that individuals with incident AD shared similar baseline features to individuals with incident ADVD, except that PAM-AMA was significantly lower in AD (inc. AD: 12.4 AMA-units; free of dementia: 12.9 AMA-Units; *p* = 0.045), and MR-proADM did not differ significantly when compared to dementia-free individuals (Fig. 2A and C, respectively). In individuals with incident VD, current smoking, CMD and CVD were more pronounced. In this group, bio-ADM and PAM-AMA did not differ from dementia-free subjects, but the MR-proADM levels were significantly higher and ADM-ratio was significantly lower (supplementary table S1B, Fig. 2A-D).

### Dementia risk in relation to plasma levels of bio-ADM, MR-proADM, ADM-ratio and PAM-AMA

In all continuous Cox models, biomarkers were inverse log-transformed prior to analysis and all HRs are therefore expressed per 1-SD decrease, such that HR > 1 consistently indicates higher risk associated with lower biomarker levels. We investigated the associations of PAM-AMA with incident dementia outcomes using continuous modelling in univariable and multivariable Cox proportional hazards analyses. The results are summarised in Fig. 2E and Table 2. In unadjusted analyses, low levels of PAM-AMA were significantly associated with an elevated risk of incident AD (Hazard Ratio per 1 SD (HR_AD_): 2.22; 95% confidence interval (CI) 1.26–3.90; *p* = 0.006), but not with incident VD (HR_VD_: 0.99; 0.42–2.31; *p* = 0.97). In the multivariable-adjusted Cox proportional hazard model, PAM-AMA remained significantly associated with incident AD (aHR_AD_: 2.30; 1.31–4.02; *p* = 0.004) and further remained a robust predictor after including the body mass index (BMI) in the multivariable-adjusted Cox proportional hazard model (aHR_AD_: 2.04; 1.17–3.53; *p* = 0.01). No significant association of PAM-AMA with incident VD was observed in adjusted analyses (Fig. 2E and Table 2).

**Table 2:** Associations between biomarkers (bio-ADM, MR-proADM, ADM-ratio, PAM-AMA) and dementia risk: Univariable and multivariable-adjusted Cox regression analysis

Biomarkers	Analysis 1: univariable cox proportional regression				Analysis 2: multivariable-adjusted cox proportional regression		Analysis 3: multivariable-adjusted cox proportional regression with BMI as additional covariate	
	Unadjusted HR (95%CI)	*p* value	LR-Chi^2^ (LR-*p* value)	C-Index (95% CI)	adjusted HR (95%CI)	*p* value/q value	adjusted HR (95%CI)	*p* value/q value
Type of dementia (population size/number of events)								
Incident Alzheimer ‘s Dementia (*n* = 4543/*n* = 205)								
PAM-AMA	2.22 (1.26–3.90)	0.006	7.48 (0.006)	0.55 (0.50–0.59)	2.30 (1.31–4.02)	0.004/0.01	2.04 (1.17–3.53)	0.01/0.03
MR-proADM	0.76 (0.45–1.30)	n.s. (0.32)	0.97 (0.32)	0.53 (0.48–0.57)	1.58 (0.86–2.91)	n.s. (0.14)/n.s. (0.14)	0.97 (0.51–1.84)	n.s. (0.93)/n.s. (0.93)
bio-ADM	1.53 (1.10–2.13)	0.01	6.43 (0.01)	0.57 (0.52–0.61)	1.62 (1.13–2.33)	0.008/0.01	1.16 (0.80–1.67)	n.s. (0.43)/n.s. (0.65)
ADM-ratio	2.01 (1.36–3.00)	0.0005	12.96 (0.0003)	0.59 (0.55–0.63)	1.51 (1.02–2.24)	0.04/NA	1.19 (0.81–1.75)	n.s. (0.38)/NA
PAM-AMA x ADM-ratio interaction	2.08 (1.50–2.88)	< 0.0001	18.66 (< 0.0001)	0.61 (0.57–0.65)	1.73 (1.25–2.40)	0.001/NA	1.43 (1.02–1.9)	0.03/NA
Incident Vascular Dementia (*n* = 4543/*n* = 93)								
PAM-AMA	0.99 (0.42–2.31)	n.s. (0.97)	0.01 (0.97)	0.51 (0.45–0.58)	1.26 (0.55–2.90)	n.s. (0.58)/n.s. (0.58)	1.22 (0.53–2.81)	n.s. (0.63)/n.s. (0.65)
MR-proADM	0.18 (0.09–0.35)	< 0.0001	21.85 (< 0.0001)	0.63 (0.58–0.69)	0.68 (0.29–1.60)	n.s. (0.38)/n.s. (0.57)	0.54 (0.23–1.30)	n.s. (0.17)/n.s. (0.51)
bio-ADM	0.97 (0.62–1.53)	n.s. (0.89)	0.018 (0.89)	0.50 (0.44–0.57)	1.32 (0.80–2.10)	n.s. (0.28)/n.s. (0.57)	1.13 (0.67–1.92)	n.s. (0.65)/n.s. (0.65)
ADM-ratio	2.56 (1.41–4.64)	0.002	10.23 (0.001)	0.59 (0.52–0.65)	1.65 (0.94–2.90)	n.s. (0.08)/NA	1.47 (0.83–2.63)	n.s. (0.19)/NA
PAM-AMA x ADM-ratio interaction	1.88 (1.16–3.05)	0.01	6.94 (0.009)	0.56 (0.49–0.62)	1.52 (0.95–2.43)	n.s. (0.08)/NA	1.39 (0.86–2.25)	n.s. (0.17)/NA

The HRs per 1 SD are shown with 95% CI. The HRs are presented either in an unadjusted form (Analysis 1) or after adjustment for age, sex, SBP, AHT, smoking, HDL, and prior history of cardiometabolic disease (Analysis 2). Analysis 3 equals to analysis 2 after inclusion of BMI as a covariate. In multivariable-adjusted analyses 2 and 3, p-values for the co-primary markers PAM-AMA, MR-proADM and bio-ADM were corrected for multiple testing using the Benjamini–Hochberg false discovery rate (FDR) method, applied separately for each endpoint and model. Resulting FDR adjusted p-values are shown as q-values. ADM-ratio and PAM-AMA x ADM-ratio interaction were considered exploratory and were not included in the FDR correction (q-value shown as NA (not applicable)). All HRs are expressed per 1-SD decrease in the respective biomarker following inverse log-transformation, such that HR > 1 indicates higher risk associated with lower biomarker levels

*Bio-ADM* Bioactive Adrenomedullin, *MR-proADM* Mid-regional pro-Adrenomedullin, *ADM-ratio* Molar ratio of bio-ADM/MR-proADM, *PAM-AMA* Peptidylglycine-alpha amidating monooxygenase amidating activity, *HR* Hazard ratio, *CI* Confidence interval, *C-Index* Concordance index, *LR-Chi*^*2*^ Likelihood-Ratio Chi^2^, *n.s.* Not significant

As the next step we investigated the association of bio-ADM, a product of PAM catalysed amidation, with incident AD and incident VD, alongside with MR-proADM and ADM-ratio. In unadjusted continuous analyses, lower levels of bio-ADM were significantly associated with an increased risk of incident AD (HR_AD_: 1.53; 1.10–2.13; *p* = 0.01) but not incident VD (HR_VD_: 0.97; 0.62–1.53; *p* = 0.89). In contrast, lower MR-proADM levels were significantly associated with a reduced risk of incident VD (HR_VD_: 0.18; 0.09–0.35; *p* < 0.0001), while no significant association was observed with incident AD (HR_AD_: 0.76; 0.45–1.30; *p* = 0.32). A lower ADM-ratio was significantly associated with an increased risk of incident AD (HR_AD_: 2.01; 1.36–3.00; *p* = 0.0005) and incident VD (HR_VD_: 2.56; 1.41–4.64; *p* = 0.002). Model fit comparisons using the Likelihood-Ratio (LR) Chi^2^ statistic demonstrated that ADM-ratio provided a stronger model fit for incident AD (LR-Chi^2^_ADM-ratio_: 12.96) than either biomarker alone (LR-Chi^2^_MR-proADM_: 0.97 and LR-Chi^2^_bio-ADM_:6.43) (Fig. 2E and Table 2), indicating that a combination of both markers adds additional risk-relevant information. For incident VD, MR-proADM yielded the highest LR-Chi^2^ value (21.85), suggesting a better performance relative to the ADM-ratio (10.23) and bio-ADM (0.018). In the multivariable-adjusted Cox proportional hazard model, no biomarkers showed a statistically significant association with incident VD (Fig. 2E and Table 2). Associations for lower bio-ADM levels with incident AD remained robust (aHR_AD_: 1.62; 1.13–2.33; *p* = 0.008) (Fig. 2E and Table 2). Notably, after multivariable adjustment the direction of the MR-proADM association with incident AD reversed without reaching statistical significance. For incident VD, the unadjusted MR-proADM association attenuated substantially after adjustment (aHR: 0.68; 0.29–1.60; n.s.), while the direction remained consistent. The ADM-ratio also remained significantly associated with incident AD (aHR_AD_: 1.51; 1.02–2.24; *p* = 0.04).

After applying Benjamini–Hochberg FDR (BH-FDR) correction across the co-primary biomarkers PAM-AMA, MR-proADM and bio-ADM in multivariable-adjusted models, PAM-AMA and bio-ADM remained significant predictors (Table 2, analysis 2).

Among the analysed markers, low levels of PAM-AMA indicated the highest risk in the multivariable-adjusted analysis (Fig. 2E and Table 2). However, other than PAM-AMA, the inclusion of the body mass index (BMI) in the multivariable-adjusted Cox proportional hazard model, confounded the risk association of bio-ADM and ADM-ratio with incident AD outcome (Fig. 2E and Table 2). PAM-AMA remained significant after BH-FDR correction (Table 2, analysis 3).

Additional adjustment for APOE ε4 status did not substantially change the described results, suggesting that ADM maturation pathway biomarkers are largely independent of APOE ε4 carrier status (Fig. 2E—light grey and supplementary table S2). However, after BH-FDR correction, the PAM-AMA association with incident AD in the BMI-adjusted model lost statistical significance (q = 0.11, supplementary table S2, analysis 3).

Due to the observed BMI dependence of bio-ADM, MR-proADM and ADM-ratio, we performed subgroup analyses stratified by BMI < 30 and BMI ≥ 30. The chosen cut-off of BMI ≥ 30 kg/m^2^ was applied to stratify participants into obese and non-obese groups, consistent with the World Health Organization definition of obesity [50]. In the BMI < 30 group, both unadjusted and adjusted results for all biomarkers (supplementary figure S1A, supplementary table S3A) closely mirrored the findings from the full cohort (as shown in Fig. 2E). However, in this subgroup, the unadjusted associations for bio-ADM with inc. AD was not significant (HR_AD_: 1.41; 0.97–2.04; *p* = 0.07). Additionally, the adjusted ADM-ratio association with incident AD lost significance (aHR_AD_: 1.38; 0.97–2.09; *p* = 0.12) while its association with incident VD became borderline significant (aHR_VD_: 1.92; 1.02–3.63; *p* = 0.05). In the BMI ≥ 30 subgroup the number of events was relatively low with *n* = 21 and *n* = 15 for AD and VD, respectively. However, only low levels of MR-proADM were associated with a reduced risk of incident AD in the unadjusted model (HR_AD_: 0.12; 0.03–0.49; *p* = 0.003) (supplementary figure S1B and supplementary table S3B). In the fully-adjusted model, no biomarker in the BMI ≥ 30 subgroup showed a statistically significant association with incident AD or VD (supplementary figure S1B and supplementary table S3B).

Given the mechanistic links between bio-ADM, MR-proADM, and PAM-AMA in the biosynthetic pathway (Fig. 1), we tested an interaction model combining ADM-ratio and PAM-AMA (interaction term (IT)-PAMxRatio). In unadjusted analyses, lower levels of the interaction term (IT) were associated with increased risk across both dementia types (HR_AD_: 2.08; 1.50–2.88; *p* < 0.0001; HR_VD_: 1.88; 1.16–3.05; *p* = 0.01) (Fig. 2E and Table 2). Despite this, LR-Chi^2^-based model fit comparisons did not demonstrate added risk-relevant value beyond the combination of individual components. While in incident AD the IT combined information content of PAM-AMA and ADM-ratio (LR-Chi^2^_IT-AD_: 18.66 vs. LR-Chi^2^_ADM-ratio-AD_: 12.96 + LR-Chi^2^_PAM-AD_: 7.48), it showed a weaker model fit in incident VD when compared to the ADM-ratio (LR-Chi^2^_IT-VD_: 6.94 vs. LR-Chi^2^_ADM-ratio-VD_: 10.23 + LR-Chi^2^_PAM-VD_: 0.01) (Fig. 2E and Table 2). The IT remained nevertheless a robust predictor for incident AD in adjusted models, even after adjusting for BMI or APOE ε4 (supplementary table S2). In BMI-stratified analyses, the IT remained significant in the BMI < 30 group but not in the BMI ≥ 30 group (supplementary figures S1A and S1B and supplementary tables S3A and S3B).

To examine whether early, undiagnosed dementia cases influenced baseline biomarker levels, we performed reverse-causation sensitivity analyses excluding dementia cases occurring within the first two years of follow-up. Across all biomarkers, the direction and magnitude of the associations were consistent with the primary continuous models. The associations of the analysed markers showed comparable effect sizes with expected reductions in statistical precision due to fewer events. After BH-FDR correction across co-primary markers, the associations of PAM-AMA and bio-ADM with incident AD lost significance. The overall association pattern did not suggest a systematic early-disease bias. Full results are provided in supplementary tables S4A, S4B and S4C. To strengthen AD specific interpretation of continuous analyses, we performed a sensitivity analysis by restricting the dataset to individuals with incident AD. Exclusion of all incident VD events, including mixed ADVD cases, did not attenuate the AD specific associations observed in primary analyses. Across all analysed markers, the pattern of associations with incident AD was comparable to that observed in continuous analyses presented in Table 2 and supplementary table S3A. Effect estimates for PAM-AMA were slightly higher in the pure AD sensitivity analysis compared with the primary models. Numerical results of the sensitivity analysis for the full cohort and in the BMI < 30 group are provided in supplementary table S4D. Finally, bootstrap-based internal validation indicated minimal optimism of concordance estimates across continuous AD models (supplementary table S4E).

### Stratified risk assessment of all-cause dementia and Alzheimer’s dementia using biomarker quartiles

Based on the data from Fig. 2A-F, Table 2, figure S1 and tables S2-4, low levels of all analysed biomarkers, except MR-proADM, were associated with increased risk of incident AD in individuals with BMI < 30 after adjusting for common dementia risk-factors. For incident VD, ADM-ratio and IT emerged as predictors. All markers were independent of APOE genotype.

Further subgroup analyses confirmed that all markers, apart from PAM-AMA, were substantially influenced by BMI. In individuals with BMI ≥ 30, no biomarker retained a statistically significant association with any dementia outcome after adjustment. In contrast, aHRs per 1SD in the BMI < 30 group were largely consistent with those from the total cohort, underscoring the robustness of these associations in normal-to-overweight individuals.

**Fig. 3:**
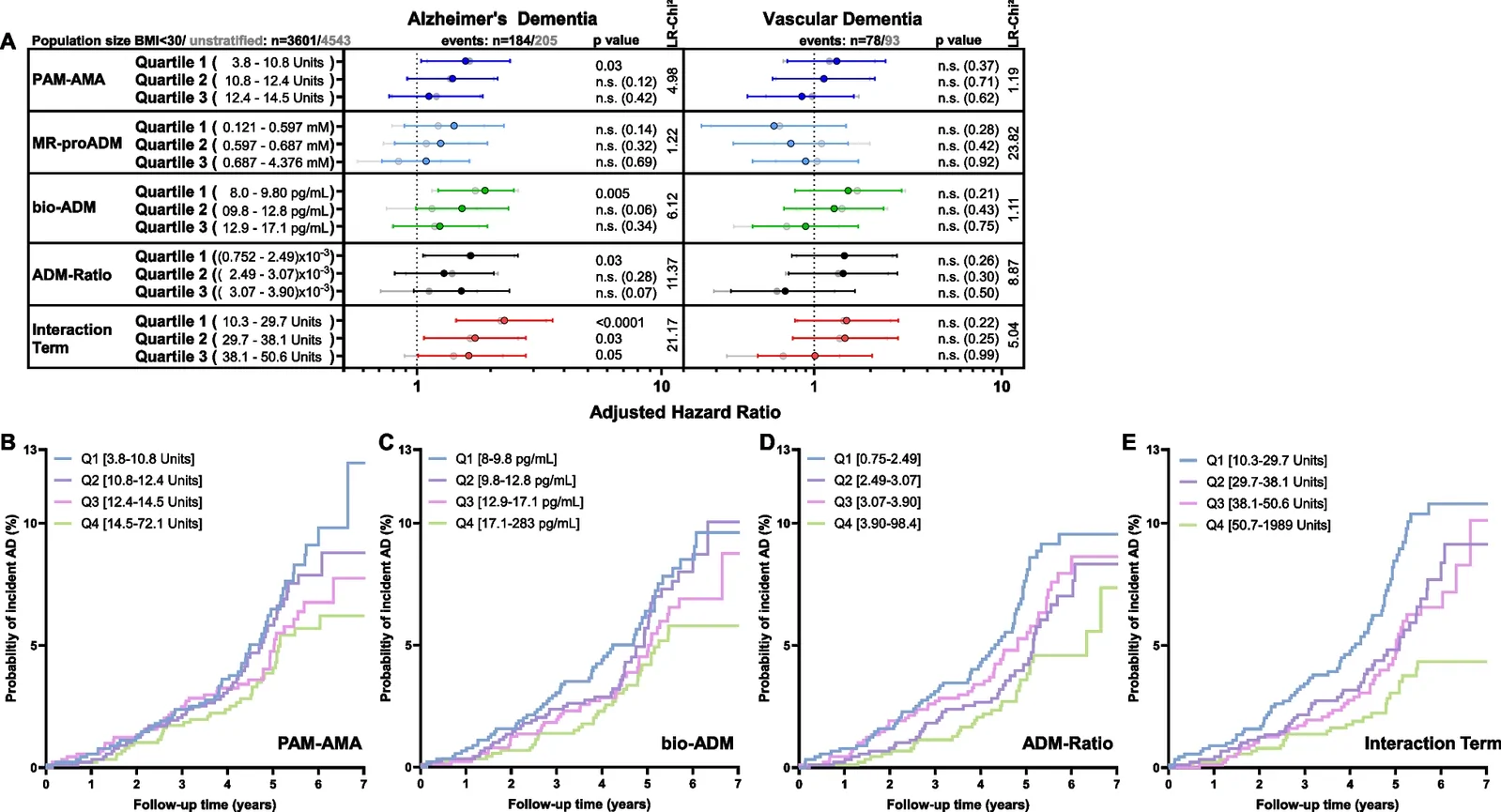
**A** Forest plots of adjusted Hazard Ratios for the biomarkers bio-ADM, MR-proADM, the ADM-ratio, PAM-AMA and the Interaction Term after stratification by BMI (BMI < 30). The HRs are shown with 95% confidence intervals. The HRs are presented either after adjustment for age, sex, SBP, AHT, smoking, HDL, and prior history of cardiometabolic disease. Shown LR-Chi^2^ values are derived from the unadjusted analysis in the BMI < 30 subgroup. In addition, the aHR values of the total study cohort are shown in light grey for matters of direct comparison. Numerical values for the BMI < 30 subgroup are summarized in Table 3. Numerical values for the full cohort and the BMI ≥ 30 are summarized in supplementary tables S5—S9. Q4 (highest biomarker levels) served as the reference category; HRs > 1 therefore indicate higher risk in lower quartiles relative to the highest quartile. B-E: Kaplan–Meier (KM) plots showing cumulative incidence of AD across quartiles of PAM-AMA (**B**), bio-ADM (**C**), ADM-ratio (**D**), and the Interaction Term (**E**) in the BMI < 30 subgroup. KM plots were generated with unadjusted data. Quartiles are derived from biomarker distributions, with Q4 (highest biomarker levels) as the reference. Q1 represents the 25th percentile and Q4 the 75th percentile of the respective biomarker distribution. Survival differences were evaluated using log-rank tests

To assess the association pattern across increasing biomarker levels, suggestive of a possible dose–response relationship, categorical analyses of the data were conducted after stratification of biomarker levels into quartiles (Q1–Q4) in the BMI < 30 subgroup. aHRs were calculated with Q4 (highest biomarker levels) as the reference group and are presented in Fig. 3A, with corresponding results from the non-BMI stratified cohort shown in light grey for comparison. Full numerical results, including unadjusted models, multivariable adjusted models, and those further adjusted for BMI are provided in supplementary tables S5-S7. Table 3 and supplementary tables S8–S9 provide numerical results for unadjusted and multivariable-adjusted models after stratification by BMI.

**Table 3:** Categorical Associations between biomarkers (bio-ADM, MR-proADM, ADM-ratio, PAM-AMA) and dementia risk after stratification by quartiles: Univariable and multivariable-adjusted Cox regression in the BMI < 30 subgroup

Biomarker quartiles		Incident Alzheimer Dementia				Incident Vascular Dementia			
	*n*	Univariable Cox regression		Multivariable-adjusted Cox regression		Univariable Cox regression		Multivariable-adjusted Cox regression	
		HR (95% CI)	*p*-value	aHR (95% CI)	*p*-value	HR (95% CI)	*p*-value	aHR (95% CI)	*p*-value
PAM-AMA									
* p*-value linear trend (LR-Chi^2^/LR-*p* value)	0.03 (4.98/0.17)			0.023		0.60 (1.19/0.78)		0.28	
Q1 (3.8–10.8 Units)	901	1.55 (1.03–2.35)	0.04	1.58 (1.04–2.40)	0.03	1.12 (0.62–2.04)	n.s. (0.71)	1.32 (0.72–2.41)	n.s. (0.37)
Q2 (10.8–12.4 Units)	900	1.39 (0.91–2.13)	n.s. (0.12)	1.40 (0.91–2.14)	n.s. (0.12)	0.95 (0.51–1.78)	n.s. (0.88)	1.13 (0.60–2.11)	n.s. (0.71)
Q3 (12.4–14.5 Units)	900	1.19 (0.77–1.84)	n.s. (0.44)	1.12 (0.77–1.86)	n.s. (0.42)	0.79 (0.41–1.51)	n.s. (0.48)	0.86 (0.44–1.63)	n.s. (0.62)
Q4 (14.5–72.1 Units)	900	Reference		Reference		Reference		Reference	
MR-proADM									
* p*-value linear trend (LR-Chi^2^/LR-*p* value)	0.39 (1.22/0.75)			0.12		< 0.0001 (23.82/< 0.0001)		0.23	
Q1 (0.121–0.597 nM)	901	0.83 (0.55–1.25)	n.s. (0.37)	1.42 (0.89–2.27)	n.s. (0.14)	0.21 (0.09–0.47)	< 0.0001	0.61 (0.25–1.48)	n.s. (0.28)
Q2 (0.597–0.687 nM)	900	0.84 (0.56–1.25)	n.s. (0.39)	1.25 (0.81–1.94)	n.s. (0.32)	0.33 (0.17–0.64)	0.001	0.75 (0.37–1.52)	n.s. (0.42)
Q3 (0.687–0.811 nM)	900	0.83 (0.56–1.23)	n.s. (0.36)	1.09 (0.72–1.64)	n.s. (0.69)	0.63 (0.37–1.05)	n.s. (0.08)	0.97 (0.57–1.68)	n.s. (0.92)
Q4 (0.811–4.376 nM)	900	Reference		Reference		Reference		Reference	
Bio-ADM									
* p*-value linear trend (LR-Chi^2^/LR-*p* value)	0.014 (6.12/0.11)			0.003		0.96 (1.11/0.78)		0.15	
Q1 (8–9.8 pg/mL)	901	1.64 (1.08–2.49)	0.02	1.90 (1.22–2.49)	0.005	0.90 (0.49–1.67)	n.s. (0.70)	1.52 (0.79–2.92)	n.s. (0.21)
Q2 (9.8–12.8 pg/mL)	900	1.42 (0.92–2.18)	n.s. (0.11)	1.53 (0.99–2.37)	n.s. (0.06)	0.99 (0.55–1.81)	n.s. (0.98)	1.28 (0.69–2.35)	n.s. (0.43)
Q3 (12.9–17.1 pg/mL)	900	1.20 (0.77–1.87)	n.s. (0.41)	1.24 (0.80–1.94)	n.s. (0.34)	0.74 (0.39–1.40)	n.s. (0.35)	0.90 (0.47–1.72)	n.s. (0.75)
Q4 (17.1–283 pg/mL)	900	Reference		Reference		Reference		Reference	
ADM-ratio (× 10^–3^)									
* p*-value linear trend (LR-Chi^2^/LR-*p* value)	0.005 (11.37/0.01)			0.06		0.008 (8.87/0.03)		0.10	
Q1 (0.752–2.49)	901	2.02 (1.31–3.12)	0.001	1.66 (1.06–2.59)	0.03	1.98 (1.07–3.71)	0.03	1.45 (0.76–2.76)	n.s. (0.26)
Q2 (2.49–3.07)	900	1.42 (0.89–2.25)	n.s. (0.14)	1.29 (0.81–2.06)	n.s. (0.28)	1.50 (0.78–2.89)	n.s. (0.24)	1.43 (0.73–2.77)	n.s. (0.30)
Q3 (3.07–3.90)	900	1.65 (1.05–2.59)	0.03	1.52 (0.97–2.39)	n.s. (0.07)	0.83 (0.39–1.78)	n.s. (0.63)	0.77 (0.36–1.65)	n.s. (0.50)
Q4 (3.90–98.4)	900	Reference		Reference		Reference		Reference	
Interaction-Term ADM-ratio x PAM-AMA (× 10^–3^)									
* p*-value linear trend (LR-Chi^2^/LR-*p* value)	< 0.0001 (21.17/< 0.0001)			< 0.0001		0.036 (5.04/0.17)		0.13	
Q1 (10.3–29.7 Units)	901	2.69 (1.72–4.20)	< 0.0001	2.28 (1.45–3.59)	< 0.0001	1.77 (0.93–3.35)	n.s. (0.08)	1.49 (0.79–2.80)	n.s. (0.22)
Q2 (29.7–38.1 Units)	900	1.86 (1.16–2.99)	0.01	1.73 (1.07–2.79)	0.03	1.63 (0.85–3.12)	n.s. (0.14)	1.46 (0.77–2.79)	n.s. (0.25)
Q3 (38.1–50.6 Units)	900	1.67 (1.03–2.70)	0.04	1.63 (1.01–2.65)	0.05	1.04 (0.51–2.12)	n.s. (0.92)	1.01 (0.50–2.04)	n.s. (0.99)
Q4 (50.7–1989 Units)	900	Reference		Reference		Reference		Reference	

Q1-Q4: Quartiles. The HRs are shown with 95% CI, Q4 was the reference quartile in all analyses, HRs > 1 therefore indicate higher risk in lower quartiles relative to Q4

*Bio-ADM* Bioactive Adrenomedullin, *MR-proADM* Mid-regional pro-Adrenomedullin, *ADM-ratio* Molar ratio of bio-ADM/MR-proADM, *PAM-AMA* Peptidylglycine-alpha amidating monooxygenase amidating activity, *BMI* Body-Mass Index, *HR* Hazard ratio, *CI* Confidence interval, *C-Index* Concordance index, *LR-Chi*^*2*^ Likelihood-Ratio Chi^2^, *n.s.* Not significant

PAM-AMA showed a significant association with increased risk of AD in the lowest quartile (Q1) in both the full cohort and in the BMI < 30 subgroup (Q1 aHR: 1.64; 1.10–2.42; *p* = 0.01 and aHR; 1.58; 1.04–2.40; *p* = 0.03, respectively) (Table 3 and supplementary table S6). While PAM-AMA shows a significant *p*-value for interquartile trend in incident AD, no significant graded association across higher quartiles was observed. Consistent with the continuous analysis, no significant association of PAM-AMA with incident VD was observed.

Comparably to PAM-AMA, bio-ADM and the ADM-ratio, but not MR-proADM, showed the highest association with AD-risk in the lowest quartile (Q1) (aHR_bio-ADM_: 1.90; 1.22–2.49; *p* = 0.005 aHR_ADM-ratio_: 1.66; 1.06–2.59; *p* = 0.03), accompanied by significant interquartile trends (borderline-significance for ADM-ratio, Fig. 3A and Table 3). No graded association with AD risk in higher quartiles (Q2 and Q3) was observed for PAM-AMA, bio-ADM, nor ADM-ratio. For MR-proADM, no significant association or interquartile trend was observed for incident AD in adjusted quartile models (Table 3). Consistent with continuous analyses, the direction of the MR-proADM association with incident AD reversed without reaching statistical significance (Table 3). For incident VD, MR-proADM showed an unadjusted gradient (Q1 HR_VD_: 0.21; *p* < 0.0001; Q2 HR_VD_: 0.33; *p* = 0.001), which attenuated to non-significance after multivariable adjustment (Q1 aHR: 0.61; n.s.), consistent with continuous analyses (Table 2).

The interaction term yielded a consistent risk association with incident AD among the analysed markers and significantly stratified risk across Q1 to Q3, suggesting a more continuous dose response relationship in the BMI < 30 subgroup (Q1 aHR: 2.28; 1.45–3.59; *p* < 0.0001, Q2 aHR: 1.73; 1.07–2.79; *p* = 0.03 and Q3 aHR: 1.63; 1.01–2.65; *p* = 0.05; Fig. 3A and Table 3).

Model comparison based on the LR-Chi^2^ values supports improved fit of the IT (LR-Chi^2^_IT_ = 21.17; *p* < 0.0001) over ADM-ratio (LR-Chi^2^_ADM-ratio_ = 11.37; *p* = 0.01) and PAM-AMA (LR-Chi^2^_PAM-AMA_ = 4.98; *p* = 0.17) when considered separately.

None of the biomarkers demonstrated consistent associations with VD in any adjusted quartile-based model, including the full cohort and the BMI < 30 subgroup (supplementary table S6 and Fig. 3A, Table 3).

To visualize time-to-event distributions across biomarker quartiles, Kaplan–Meier (KM) plots were generated for all biomarkers that demonstrated significant associations with dementia endpoints in the categorical analysis. Specifically, KM curves are shown for PAM-AMA, bio-ADM, ADM-ratio, and the IT, in relation to incident AD (Fig. 3B-E).

In the case of PAM-AMA, the KM curves across quartiles overlap up to approximately 4 years of follow-up. Q1 and Q2 exhibit similar trajectories, which diverge from Q3 and Q4 after 4 years of follow-up suggesting a threshold-like effect below the median (median_BMI<30_ = 12.44 PAM-Units) (Fig. 3B). Q1 shows the steepest incidence trajectory, particularly after mid-follow-up, suggesting a delayed but accelerated AD risk in individuals with low PAM activity (Fig. 3B). For bio-ADM, the KM plot (Fig. 3C) shows a modest separation between lower (Q1 and Q2) and higher quartiles. Q1 exhibits a higher cumulative incidence, while Q3 and Q4 show comparably lower incidence, indicating a threshold-like effect below the median (median _BMI<30_ = 12.84 pg/mL), with relatively stable risk above it. For the ADM-ratio, the highest AD incidence was observed in Q1 (Fig. 3D), with a clear decline in risk toward Q4. The IT shows the clearest and most distinct separation among quartiles. Q1 consistently displayed the highest cumulative incidence, while Q4 shows the lowest cumulative AD incidence. Q2 and Q3 however display similar and overlapping trajectories. This pattern supports a graded, dose–response like relationship between the combined effect of reduced PAM activity and impaired ADM conversion and dementia risk (Fig. 3E).

Reversed causation sensitivity analyses excluding dementia cases occurring within the first two years of follow-up showed directionally consistent quartile-based associations with incident AD compared with the primary categorical models, supporting robustness of the observed risk stratification (supplementary tables S10–S12).

## Discussion

This study investigated associations of circulating PAM-AMA and biomarkers of the ADM biosynthesis pathway (Fig. 1) with incident AD and VD. Combined cases of AD and VD (ADVD) were used for baseline characterisation. Among the analysed biomarkers, PAM-AMA was the strongest and most consistent predictor of incident AD, and remained significant after multivariable adjustment, including BMI and APOE ε4 as covariables. Lower bio-ADM levels were also associated with increased AD risk, while a reduced ADM-ratio and the composite IT (PAM-AMA × ADM-ratio) suggested that reduced enzymatic activity of PAM, and not a reduced pro-adrenomedullin synthesis, contributes to dementia risk. These findings propose that a reduced systemic amidation efficiency is associated with incident AD and may represent an additional pathway involved in AD pathophysiology. Absolute differences in levels of PAM-AMA, bio-ADM, and ADM-ratio were modest. However, given that these markers reflect enzymatic activity influencing peptide maturation within regulated physiological pathways, even small changes may be relevant at a population level. Additionally, in preclinical disease states substantial variations of these markers are not necessarily expected. In this context, modest differences at baseline may reflect beginning changes in physiological reserve rather than an overt dysfunction. Since no established reference ranges or prior data exist for PAM-AMA or bio-ADM in dementia, effect size interpretation relies on internal risk gradients within our study population, quartile-based patterns, and multivariable model performance rather than on defined thresholds.

Prior studies reported reduced PAM activities in CSF and brain of AD patients [23] and reduced plasmatic PAM levels in proteomic datasets across neurodegenerative disorders [24]. Unfortunately, these studies did not investigate whether reduced PAM levels translate into a reduction of amidated peptide hormone levels. Our findings link reduced circulating PAM-AMA to reduced levels of bio-ADM suggesting that potential deficits in systemic peptide amidation are associated with dementia risk. In the MPP cohort, lower plasma levels of PAM-AMA at baseline predicted incident AD, suggesting that systemic inefficiency of peptide amidation may contribute to early neurovascular vulnerability. This association was independent of APOE ε4 carrier status, which is consistent with proteomic evidence showing that plasma PAM levels are not influenced by APOE genotype [24]. This indicates that impaired amidating activity may represent an additional, APOE-independent risk-associated pathway contributing to AD. The association remained robust after adjustment for BMI, indicating that the relationship between PAM-AMA and incident AD is not confounded by metabolic status. However, obesity influenced related biomarker associations from the ADM maturation pathway (supplementary table S2).

Bio-ADM, generated by PAM-dependent amidation of ADM-Gly, is a potent endothelial maintenance hormone that regulates vascular tone, stabilizes the BBB, and exerts anti-inflammatory and neuroprotective actions [36, 37]. Previous studies showed that exogenous administration of ADM stabilizes endothelial barrier integrity, reduces inflammation, and improves outcomes in models of acute vascular injury [26]. In the present study, lower levels of bio-ADM at baseline were associated with higher risk of incident AD, but not VD. Chronically reduced bio-ADM may therefore lead to impaired vascular homeostasis and indicate an inability to maintain protective signalling before clinical dementia becomes evident. This interpretation is consistent with the progressive, microvascular nature of AD [51], in contrast to the acute and heterogeneous etiologies typical for VD [52, 53], often triggered by acute vascular insults [53], during which circulating bio-ADM levels increase as an injury response [54]. In this study, AD incidence in the BMI < 30 subgroup was highest among participants in the lowest bio-ADM quartile, suggesting reduced bio-ADM signalling leading to a chronic deficiency in vascular maintenance. However, the absence of statistically significant associations with incident VD after multivariable adjustment should be interpreted cautiously. The VD subgroup comprised only 93 incident events, resulting in limited statistical power and wide confidence intervals. Notably, adjusted effect estimates for PAM-AMA and bio-ADM remained directionally consistent with the AD findings, suggesting that the VD analyses are inconclusive rather than evidence against a role of the PAM-ADM pathway in vascular cognitive impairment.

MR-proADM, a stable but biologically inactive ADM-fragment (Fig. 1), was elevated at baseline in AD and VD (Table S1B), consistent with prior literature [44]. In both continuous and categorical unadjusted analyses, higher MR-proADM levels were directionally consistently associated with increased risk for both AD and VD. For incident VD, this association was significant in unadjusted models but lost statistical significance after multivariable adjustment (Tables 2 and 3), consistent with previous MPP analyses [44], potentially reflecting confounding by cardiometabolic burden disproportionately elevated in the VD subgroup (Table S1B). For incident AD, the unadjusted associations were statistically not significant but directionally consistent with the baseline observation. After multivariable adjustment, the direction reversed toward numerically higher AD-risk at lower MR-proADM levels, without reaching statistical significance. Although the direction of the MR-proADM association with incident AD reversed after multivariable adjustment, this pattern is not contradictory when considered in the context of the bio-ADM/MR-proADM ratio: despite elevated MR-proADM levels at baseline (Tables 1 and S1), bio-ADM was simultaneously reduced in incident AD, resulting in a reduced ADM-ratio that remained significantly associated with AD risk after adjustment.

The inverse relationship between MR-proADM and bio-ADM supports the interpretation that reduced bio-ADM levels may reflect inefficient amidation of ADM-Gly rather than decreased pro-ADM synthesis, which is reflected by the ADM-ratio.

The molar ratio of bio-ADM to MR-proADM (ADM-ratio) combines both peptide amidation and precursor accumulation. A lower ratio may reflect reduced amidation efficiency or an uncoupling between pro-ADM synthesis and amidation. Such uncoupling of precursor and active peptide was observed in acute disease states, where a rapid elevation of ADM-Gly and delayed PAM release create an ‘amidation bottleneck’ resulting in an unfavourable reduced ADM-ratio [55, 56]. In our analyses, a reduced ADM-ratio was significantly associated with higher AD risk and provided better model performance than either individual marker (Fig. 2, Table 2). These findings suggest that the ADM-ratio may reflect the functional state of peptide amidation.

To combine enzymatic PAM activity, peptide amidation and precursor accumulation into a single parameter, we tested a composite interaction term (PAM-AMA × ADM-ratio). Although the global model fit was similar to ADM-ratio alone (Fig. 2E, Table 2), the IT consistently predicted AD risk in multivariable models and demonstrated a graded risk stratification in quartile-based analyses, particularly in individuals with BMI < 30 (Fig. 3A, Table 3). This pattern indicates that the IT may capture a subgroup with particularly high vulnerability to AD by connecting systemic amidation deficiency with precursor accumulation and reduced amidated product formation.

Across all analyses, sensitivity testing confirmed robustness of main findings. Reverse-causation models indicated that the described findings are unlikely to reflect prodromal disease. Consistent results were observed after restriction of the data set to AD as the only endpoint and correction for multiple testing and internal bootstrap validation suggest minimal influence of overfitting.

BMI confounded associations of bio-ADM and ADM-ratio with incident AD: in obese individuals (BMI ≥ 30), no associations remained after adjustment (Fig. 2E, Table 2). Chronic low-grade inflammation and oxidative stress in adipose tissue upregulate ADM expression as a compensatory vasoprotective response [57]. Consequently, elevated baseline ADM in obese individuals may confound the detection of amidation-related deficits, reducing the discriminative value of bio-ADM and ADM-ratio. Additionally, event numbers in this subgroup were small (n_AD_ = 21 and n_VD_ = 15), limiting statistical power. Nonetheless, the consistent pattern across individuals with BMI < 30 indicates that amidation deficits are most evident in metabolically less inflamed environments without compensatory ADM upregulation [52]. Our observation aligns with prior reports describing a reduced AD risk in subjects with a higher late-life BMI [58].

Mechanistically, PAM-dependent amidation is essential for the activity of multiple vasoactive and neuroprotective peptides that exhibit vasodilatory, anti-inflammatory, and neuroprotective effects, stabilize BBB integrity and modulate amyloid clearance in preclinical models [12, 59–62]. Deficient amidation may influence multiple protective signalling pathways and therefore amplify vascular and neuronal vulnerability.

Experimental studies support that PAM can contribute to peptide amidation in circulation [45, 56, 63, 64]. Further, heterozygous or conditional knockdowns of PAM were shown to affect cognitive phenotypes in animal models [65–67]. These data underscore that systemic amidation efficiency is not exclusively a passive reflection of tissue PAM expression, but a dynamic, biologically active process that may influence neurovascular health.

Our findings align with the vascular hypothesis of AD, which proposes that cerebrovascular dysfunction is an early and critical event in AD development [5]. Bio-ADM is highly expressed in brain microvascular endothelial cells [68] (BMVECs) and is a key regulator of vascular tone and BBB integrity [37, 68]. Bio-ADM promotes endothelial survival and barrier integrity [37, 68–70]. Therefore, reduced PAM-AMA may negatively influence ADM amidation leading to the observed reduced bio-ADM levels. Given the role of bio-ADM in endothelial function and BBB regulation, reduced bio-ADM signalling may lead to endothelial fragility, BBB disruption, and impaired amyloid clearance, processes observed in early AD pathology [71]. These findings are compatible with the vascular hypothesis of AD, although a causality between impaired systemic amidation and neurodegenerative processes cannot be proven with the present data set.

Given the coexistence of precursor and amidated peptides in plasma [11], the observation that systemic amidation efficiency is associated with AD risk raises the question of whether PAM activity represents a modifiable factor in neurovascular homeostasis. Preclinical data demonstrate that PAM activity can be pharmacologically modulated and stabilized [63], providing a basis for experimental investigation. Whether a modulation of PAM activity translates into effects on endothelial function, BBB integrity or cognitive outcomes remains to be established. Future studies in appropriate preclinical models of neurovascular dysfunction should first determine whether the observed associations reflect a causal pathway before translational conclusions can be drawn.

### Strengths and limitations

This population-based study used a well-characterised cohort to investigate novel biomarkers of AD risk. The study combined enzymatic PAM activity and precursor-to-product biomarkers within the maturation pathway of ADM. However, limitations include that primary care is not covered in the SNPR and the mean follow-up of 4.75 years is relatively short. This may have resulted in an underestimation of incident dementia cases and may limit the distinction between early prodromal and established disease states. Dementia diagnoses were based on national registry data without supporting AD biomarker confirmation and followed DSM-IV-based clinical criteria and may be associated with reduced diagnostic specificity and phenotypic resolution. No amyloid-PET, CSF Aβ/tau biomarker data were available to confirm AD pathology, potentially leading to misclassification of mixed or non-AD dementia cases as AD. The reliance on a predominantly European population in the studied cohort may limit generalisability to other ethnic groups. Based on selection criteria, the analysed cohort showed a higher proportion of men and modest differences in cardiometabolic comorbidity compared with the remaining MPP-RES population. Although these factors were accounted for in multivariable analyses where appropriate, some degree of selection bias cannot be excluded. The subgroup analyses, particularly the BMI ≥ 30 subgroup, had limited event numbers and should be interpreted as exploratory. The used PAM-AMA assay was performed under optimal concentrations of the cofactors Copper and Ascorbate [45] and therefore reflects total circulating PAM concentration rather than in vivo catalytic activity. The proposed mechanistic links between reduced PAM activity, diminished amidated hormone levels, and dementia risk rely on observational associations and interpretative inference rather than demonstrated causality. The study findings originate from a single study cohort and therefore require validation in an independent cohort with longitudinal design. Internal bootstrap validation cannot substitute for external replication, which remains a key priority for future work. The presented conclusions and hypotheses remain provisional and require targeted experimental validation to confirm the proposed biological pathway.

### Conclusion

In summary, this study identified reduced plasma amidating activity of PAM and lower circulating bio-ADM levels as independent risk markers for incident AD but not VD. The observed associations suggest that reduced PAM activity and lower bio-ADM levels may reflect impaired peptide hormone amidation in individuals who later develop AD. Given that PAM activates multiple neurovascular and neuroprotective peptides, its relevance may extend beyond vascular dysfunction to other core features of AD pathology. These findings are hypothesis-generating and given the observational design and absence of biomarker-confirmed AD diagnoses and neuroimaging data, they should be interpreted as observational associations requiring replication in cohorts with biomarker-validated dementia diagnoses. Whether PAM represents a modifiable target remains to be established. Therefore, future preclinical studies should investigate whether modulation of circulating PAM activity influences neurovascular function, representing a necessary first step before translational conclusions can be drawn.

## Supplementary Information

Supplementary Material 1.

## Data Availability

The dataset analysed during the current study is not publicly available but is available from the corresponding author on reasonable request and subject to appropriate ethical and data protection approvals and institutional authorization by principal investigators of the Malmö Preventive Project.
