# Supplementary material for: Impaired peptide amidation and risk of incident dementia in the Malmoe preventive project cohort
Source: Alzheimers Res Ther. 2026 Sep 28;18:217. doi: 10.1186/s13195-026-02173-7 (PMC13621642; doi:10.1186/s13195-026-02173-7)
Supplement: Supplementary file 1 [file 13195_2026_2173_MOESM1_ESM.docx]

**Supplementary Information** **for the manuscript**:

Impaired peptide amidation and risk of incident dementia in the Malmoe preventive project cohort.

**Abbreviations used**

**ADM** – total Adrenomedullin irrespective of amidation status

**ADM-Gly** – C-terminally glycine extended Adrenomedullin

**AMA** – Amidating Activity

**bio-ADM** – C-terminally amidated bioactive Adrenomedullin
**MR-proADM** – Mid-regional pro-Adrenomedullin

**PAM** – Peptidylglycine α-Amidating Monooxygenase

**CMD** – Cardiometabolic Diseases
**CVD** – Cardiovascular Diseases

**BP** – Blood Pressure

**AHT** – Antihypertensive Treatment

**LDL** - low-density lipoprotein

**HDL** - high-density lipoprotein,

**IQR** – Interquartile Range

**LOD** – Limit of Detection

**LOQ** – Limit of Quantification

**moAb** – Monoclonal Antibody

**BMI** – Body Mass Index

**APOE** – Apolipoprotein E

**AD** – Alzheimer’s Dementia

**VD** – Vascular Dementia

**Q** – Quartile

Supplementary figure S1: Forest Plots of Hazard Ratios per 1 SD for the biomarkers PAM-AMA, MR-proADM, bio-ADM, the ADM-Ratio and the Interaction Term after stratification by BMI (BMI<30 - A and BMI≥30 - B). The HRs are shown with 95% confidence intervals. The HRs are presented either in an unadjusted form (unadjusted) or after adjustment for age, sex, SBP, AHT, smoking, HDL, and prior history of cardiometabolic disease (adjusted). Each analysis was performed for the indications incident Alzheimer’s (AD) and incident Vascular dementia (VD). Numerical values are summarized in supplementary tables S3A and S3B.

Supplementary table S1A: Characteristics of the analysed MPP study population (n=4543) in comparison to the remaining MPP-RES population.

| **Characteristics** | **Selected Subcohort**  **(n= 4543)** | **Selected Subcohort free of Dementia**  **(n= 4259)** | **Remaining MPP-RES cohort**  **(n=13567)** | **p-Value 1 (Selected vs MPP-RES)** | **p-Value 2 (Selected dementia-free vs MPP-RES)** | **p-Value 3 (Selected vs. selected dementia-free)** |
| --- | --- | --- | --- | --- | --- | --- |
| Age (years) | 69.8 +/- 6.2 | 69.5 +/- 6.2 | 68.4 +/- 5.6 | < 0.001 | < 0.001 | 0.041 |
| Sex, (% male) | 70.2 | 71.0 | 61.1 | < 0.001 | < 0.001 | n.s. |
| Current smoking, (%) | 18.4 | 18.8 | 16.6 | 0.006 | 0.001 | n.s. |
| Systolic BP (mmHg) | 145.5 +/- 20.3 | 145.7 +/- 20.3 | 144.6 +/- 19.9 | 0.008 | 0.003 | n.s. |
| Diastolic BP (mmHg) | 83.6 +/- 10.8 | 83.8 +/- 10.8 | 83.5 +/- 10.5 | n.s | n.s | n.s |
| Antihypertensive treatment, (%) | 39.3 | 38.9 | 37.8 | n.s | n.s | n.s |
| Plasma LDL (mM) | 3.6 +/- 1 | 3.6 +/- 1 | 3.6 +/- 1 | n.s | n.s | n.s |
| Plasma HDL (mM) | 1.38 +/- 0.4 | 1.37 +/- 0.4 | 1.42 +/- 0.4 | < 0.001 | < 0.001 | n.s. |
| Plasma cholesterol (mM) | 5.6 +/- 1.1 | 5.6 +/- 1.1 | 5.6 +/- 1.1 | 0.003 | 0.002 | n.s. |
| Glucose (mM) | 5.9 +/- 1.5 | 5.9 +/- 1.5 | 5.8 +/- 1.4 | n.s | n.s | n.s |
| BMI | 27.1 +/- 4.2 | 27.2 +/- 4.2 | 27.2 +/- 4.1 | 0.043 | n.s | n.s |
| Plasma triglycerides (mM) | 1.2 +/- 0.6 | 1.2 +/- 0.6 | 1.3 +/- 0.9 | <0.001 | 0.004 | n.s. |
| History of CMD (%) | 27.9 | 27.6 | 25.2 | < 0.001 | 0.002 | n.s. |
| History of CVD (%) | 17.4 | 16.8 | 14.1 | < 0.001 | < 0.001 | n.s. |
| Stroke prevalence (%) | 4.3 | 4.0 | 3.3 | 0.001 | 0.03 | n.s. |

BP: blood pressure, LDL: low-density lipoprotein, HDL: high-density lipoprotein, CMD: cardiometabolic disease; CVD: cardiovascular disease, Data are presented as mean +/- standard deviation (SD) or as percentage of the respective subgroup. Differences in continuous variables were tested using independent t-tests, categorical variables were compared with Pearson’s Chi-square test.

Supplementary table S1B: Characteristics of the analysed MPP study population (n=4543) after stratification for dementia subtypes.

| **Characteristics** | **Alzheimer’s Dementia**  **(n= 205)** | **Vascular Dementia**  **(n= 93)** | **Free of Dementia (n=4259)** | **p-Value** |
| --- | --- | --- | --- | --- |
| Age (years) | 73.5 +/- 4.1 | 75.0 +/- 4.8 | 69.5 +/- 6.2 | < 0.0001 |
| Sex, (% male) | 53.2 | 69.9 | 71.0 | < 0.0001 |
| Current smoking, (%) | 9.3 | 20.4 | 18.8 | 0.02 |
| Systolic BP (mmHg) | 142.0 +/- 18.9 | 148.1 +/- 21.7 | 145.7 +/- 20.3 | 0.03 |
| Diastolic BP (mmHg) | 81.2 +/- 10.1 | 83.7 +/- 11.1 | 83.8 +/- 10.8 | 0.005 |
| Antihypertensive treatment, (%) | 39.0 | 59.1 | 38.9 | 0.0001 |
| Plasma LDL (mM) | 3.7 +/- 1 | 3.4 +/- 1 | 3.6 +/- 1 | n.s. |
| Plasma HDL (mM) | 1.48 +/- 0.43 | 1.40 +/- 0.46 | 1.37 +/- 0.4 | 0.0001 |
| Plasma cholesterol (mM) | 5.7 +/- 1.1 | 5.3 +/- 1.2 | 5.6 +/- 1.1 | 0.02 |
| Glucose (mM) | 5.6 +/- 1 | 5.8 +/- 1.6 | 5.9 +/- 1.5 | 0.04 |
| BMI | 25.4 +/- 3.5 | 26.1 +/- 4.1 | 27.2 +/- 4.2 | < 0.0001 |
| Plasma triglycerides (mM) | 1.2 +/- 0.6 | 1.1 +/- 0.6 | 1.24 +/- 0.63 | 0.01 |
| History of CMD (%) | 27.3 | 46.2 | 27.6 | < 0.0001 |
| History of CVD (%) | 19.0 | 40.9 | 16.8 | < 0.0001 |
| Stroke prevalence (%) | 3.9 | 18.3 | 4.0 | < 0.0001 |
| APOE ε4 homozygous (%) | 13.7 | 7.5 | 2.1 | < 0.0001 |
| APOE ε4 heterozygous (%) | 48.3 | 36.6 | 25.3 | < 0.0001 |
| APOE ε4 negative (%) | 35.1 | 54.8 | 69.1 | < 0.0001 |
| APOE status missing (%) | 2.9 | 1.1 | 3.5 | N/A |
| PAM-AMA (AMA-Units) | 12.4 +/- 3.2 | 13 +/- 3.6 | 12.9 +/- 3.3 | n.s. |
| bio-ADM (pg/mL) | 15.3 +/- 10.0 | 16.7 +/- 10.0 | 16.4 +/- 12.1 | 0.02 |
| MR-proADM (nM) | 0.77 +/- 0.22 | 0.86 +/- 0.25 | 0.75 +/- 0.2 | 0.0001 |
| ADM-Ratio (x10^-3^) | 3.3 +/- 1.9 | 3.2 +/- 1.6 | 3.7 +/- 2.7 | 0.0001 |

BP: blood pressure, LDL: low-density lipoprotein, HDL: high-density lipoprotein, CMD: cardiometabolic disease; CVD: cardiovascular disease, bio-ADM: bioactive Adrenomedullin, MR-proADM: mid-regional pro-Adrenomedullin, ADM-Ratio: molar ratio of bio-ADM/MR-proADM, PAM-AMA: peptidylglycine-alpha amidating monooxygenase amidating activity. The ApoE status was missing in n=156 of 4543 specimen. Data is presented as mean +/- standard deviation (SD) or as percentage of the respective subgroup. Differences in continuous variables were tested with ordinary ANOVA, categorical variables were compared with Fisher’s Chi-square test.

Supplementary table S2: Associations between biomarkers (bio-ADM, MR-proADM, ADM-Ratio, PAM-AMA) and dementia risk: Univariable and multivariable-adjusted Cox regression analysis including presence of APOE ε4 as covariable.

|  | **Analysis 1: univariable cox proportional regression** | | | | **Analysis 2: multivariable-adjusted cox proportional regression** | | | **Analysis 3: multivariable-adjusted cox proportional regression with BMI as additional covariate** | | |
| --- | --- | --- | --- | --- | --- | --- | --- | --- | --- | --- |
| Biomarkers | Unadjusted HR (95%CI) | p value | LR-Chi²  (LR-p value) | C-Index (95% CI) | adjusted HR (95%CI) | p value / q value | ΔaHR (w/wo APOE ε4) | adjusted HR (95%CI) | p value / q value | ΔaHR (w/wo APOE ε4 |
| **Type of dementia (population size / number of events)** | | | | | | | | | | |
| **Incident Alzheimer‘s Dementia (n=4388 / n=199)** | | | | | | | | | | |
| PAM-AMA | 1.82 (1.02 – 3.24) | 0.04 | 4.10 (0.04) | 0.54 (0.50 – 0.58) | 2.05 (1.15 – 3.64) | 0.02 / 0.045 | 0.13 | 1.82 (1.03 – 3.20) | 0.04 / n.s (0.11) | 0.12 |
| MR-proADM | 0.74 (0.43 – 1.27) | n.s. (0.27) | 1.21 (0.27) | 0.53 (0.49 – 0.57) | 1.41 (0.76 – 2.61) | n.s. (0.27) / n.s. (0.27) | -0.17 | 0.95 (0.51 – 1.78) | n.s. (0.88) / n.s. (0.88) | -0.03 |
| Bio-ADM | 1.48 (1.05 – 2.07) | 0.02 | 5.31 (0.02) | 0.56 (0.52 – 0.61) | 1.47 (1.04 – 2.09) | 0.03 / 0.045 | -0.09 | 1.11 (0.78 – 1.59) | n.s. (0.56) / n.s. (0.84) | -0.005 |
| ADM-Ratio | 1.95 (1.31 – 2.90) | 0.001 | 11.52 (0.0007) | 0.59 (0.54 – 0.63) | 1.39 (0.96 – 2.05) | n.s. (0.08) / NA | -0.04 | 1.14 (0.79 – 1.67) | n.s. (0.48) / NA | 0.007 |
| PAM-AMA x ADM-Ratio interaction | 1.91 (1.37 – 2.65) | 0.0001 | 15.52 (<0.0001) | 0.60 (0.56 – 0.64) | 1.57 (1.13 – 2.18) | 0.007 / NA | -0.006 | 1.32 (0.96 – 1.83) | n.s. (0.09) / NA | 0.02 |
| **Incident Vascular Dementia (n= 4388/ n=92)** | | | | | | | | | | |
| PAM-AMA | 1.04 (0.44 – 2.44) | n.s. (0.94) | <0.01 (0.93) | 0.49 (0.43 – 0.56) | 1.36 (0.59 – 3.17) | n.s. (0.47) / n.s. (0.48) | 0.02 | 1.32 (0.59 – 3.05) | n.s. (0.52) / n.s. (0.73) | 0.03 |
| MR-proADM | 0.17 (0.09 – 0.33) | <0.0001 | 22.96 (<0.0001) | 0.64 (0.58 – 0.70) | 0.62 (0.26 – 1.46) | n.s. (0.27) / n.s (0.48) | -0.02 | 0.51 (0.21 – 1.21) | n.s. (0.13) / n.s. (0.38) | -0.001 |
| Bio-ADM | 0.96 (0.61 – 1.51) | n.s. (0.85) | 0.04 (0.85) | 0.50 (0.44 – 0.57) | 1.26 (0.77 – 2.09) | n.s. (0.36) / n.s. (0.48) | -0.02 | 1.10 (0.65 – 1.86) | n.s. (0.73) / n.s. (0.73) | 0.001 |
| ADM-Ratio | 2.56 (1.41 – 4.66) | 0.002 | 10.20 (0.001) | 0.59 (0.52 – 0.66) | 1.61 (0.92 – 2.82) | n.s. (0.10) / NA | -0.007 | 1.45 (0.82 – 2.59) | n.s. (0.20) / NA | 0.008 |
| PAM-AMA x ADM-Ratio interaction | 1.92 (1.18 – 3.12) | 0.008 | 7.30 (0.007) | 0.56 (0.50 – 0.63) | 1.54 (0.96 – 2.47) | n.s. (0.07) / NA | 0.007 | 1.42 (0.88 – 2.31) | n.s. (0.15) / NA | 0.02 |

Bio-ADM: bioactive Adrenomedullin, MR-proADM: mid-regional pro-Adrenomedullin, ADM-Ratio: molar ratio of bio-ADM/MR-proADM, PAM-AMA: peptidylglycine-alpha amidating monooxygenase amidating activity. HR: hazard ratio, CI: confidence interval, C-Index: concordance index, LR-Chi²: Likelihood-Ratio Chi², n.s.: not significant. The HRs per 1 SD are shown with 95% CI. The HRs are presented either in an unadjusted form (Analysis 1) or after adjustment for age, sex, SBP, AHT, smoking, HDL, APOE ε4 status and prior history of cardiometabolic disease (Analysis 2). Analysis 3 equals to analysis 2 after inclusion of BMI as a covariate. ΔaHR (w/wo ApoE4) indicates the absolute change in adjusted hazard ratio after adding APOE ε4 status to the multivariable model (APOE ε4 -adjusted aHR minus non– APOE ε4 -adjusted aHR). In Analyses 2 and 3, p-values for the co-primary markers PAM-AMA, MR-proADM and bio-ADM were corrected for multiple testing using the Benjamini-Hochberg false discovery rate (FDR) method, applied separately for each endpoint and model. Resulting FDR adjusted p-values are shown as q-values. ADM-Ratio and PAM-AMA x ADM-Ratio interaction were considered exploratory and were not included in the FDR correction (q-value shown as NA (not applicable)).

Supplementary table S3A: Associations between biomarkers (bio-ADM, MR-proADM, ADM-Ratio, PAM-AMA) and dementia risk: Univariable and multivariable-adjusted Cox regression analysis after stratification by BMI (BMI<30).

|  | **Analysis 1 in BMI<30: univariable cox proportional regression** | | | | **Analysis 2 in BMI<30: multivariable-adjusted cox proportional regression** | |
| --- | --- | --- | --- | --- | --- | --- |
| Biomarkers | Unadjusted HR (95%CI) | p value | LR Chi²  (LR-p value) | C-Index (95% CI) | adjusted HR (95%CI) | p value / q value |
| **Type of dementia (population size / number of events)** | | | | | | |
| **Incident Alzheimer‘s Dementia (n=3601 / n=184)** | | | | | | |
| PAM-AMA | 2.29 (1.27 – 4.13) | 0.006 | 7.49 (0.006) | 0.55 (0.50 – 0.59) | 2.23 (1.25 – 3.99) | 0.007 / 0.02 |
| MR-proADM | 0.75 (0.42 – 1.35) | n.s. (0.33) | 0.92 (0.33) | 0.53 (0.49 – 0.58) | 1.70 (0.87 – 3.36) | n.s. (0.12) / n.s. (0.12) |
| Bio-ADM | 1.41 (0.97 – 2.04) | n.s. (0.07) | 3.46 (0.06) | 0.55 (0.51 – 0.59) | 1.57 (1.06 – 2.33) | 0.02 / 0.04 |
| ADM-Ratio | 1.75 (1.15 – 2.66) | 0.009 | 7.23 (0.007) | 0.57 (0.53 – 0.61) | 1.38 (0.92 – 2.09) | n.s. (0.12) / NA |
| PAM-AMA x ADM-Ratio interaction | 1.93 (1.36 – 2.75) | 0.0003 | 14.20 (0.0002) | 0.60 (0.55 – 0.64) | 1.62 (1.15 – 2.30) | 0.006 / NA |
| **Incident Vascular Dementia (n= 3601/ n=78)** | | | | | | |
| PAM-AMA | 1.04 (0.42 – 2.60) | n.s. (0.93) | 0.008 (0.92) | 0.50 (0.43 – 0.57) | 1.29 (0.52 – 3.19) | n.s. (0.58) / n.s. (0.58) |
| MR-proADM | 0.14 (0.07 – 0.28) | <0.0001 | 24.01 (<0.0001) | 0.65 (0.59 – 0.71) | 0.56 (0.21 – 1.48) | n.s. (0.25) / n.s. (0.37) |
| Bio-ADM | 0.98 (0.58 – 1.66) | n.s. (0.94) | 0.005 (0.94) | 0.51 (0.44 – 0.58) | 1.44 (0.80 – 2.58) | n.s. (0.22) / n.s. (0.37) |
| ADM-Ratio | 3.16 (1.59 – 6.29) | 0.001 | 11.78 (0.0006) | 0.60 (0.53 – 0.67) | 1.92 (1.02 – 3.63) | 0.05 / NA |
| PAM-AMA x ADM-Ratio interaction | 2.17 (1.25 – 3.75) | 0.006 | 8.19 (0.004) | 0.57 (0.51 – 0.64) | 1.71 (1.01 – 2.89) | 0.05 / NA |

Bio-ADM: bioactive Adrenomedullin, MR-proADM: mid-regional pro-Adrenomedullin, ADM-Ratio: molar ratio of bio-ADM/MR-proADM, PAM-AMA: peptidylglycine-alpha amidating monooxygenase amidating activity. BMI: Body-Mass Index, HR: hazard ratio, CI: confidence interval, C-Index: concordance index, LR-Chi²: Likelihood-Ratio Chi², n.s.: not significant. The HRs per 1 SD are shown with 95% CI. The HRs are presented either in an unadjusted form (Analysis 1) or after adjustment for age, sex, SBP, AHT, smoking, HDL, and prior history of cardiometabolic disease (Analysis 2). The data is presented for the BMI<30 subgroup. In Analysis 2, p-values for the co-primary markers PAM-AMA, MR-proADM and bio-ADM were corrected for multiple testing using the Benjamini-Hochberg false discovery rate (FDR) method, applied separately for each endpoint and model. Resulting FDR adjusted p-values are shown as q-values. ADM-Ratio and PAM-AMA x ADM-Ratio interaction were considered exploratory and were not included in the FDR correction (q-value shown as NA (not applicable)).

Supplementary table S3B: Associations between biomarkers (bio-ADM, MR-proADM, ADM-Ratio, PAM-AMA) and dementia risk: Univariable and multivariable-adjusted Cox regression analysis after stratification by BMI (BMI≥30).

|  | **Analysis 1 in BMI**≥**30: univariable cox proportional regression** | | | | **Analysis 2 in BMI**≥**30: multivariable-adjusted cox proportional regression** | |
| --- | --- | --- | --- | --- | --- | --- |
| Biomarkers | Unadjusted HR (95%CI) | p value | LR Chi²  (LR-p value) | C-Index (95% CI) | adjusted HR (95%CI) | p value / q value |
| **Incident Alzheimer‘s Dementia (n=942 / n=21)** | | | | | | |
| PAM-AMA | 1.17 (0.18 – 7.83) | n.s. (0.87) | 0.03 (0.87) | 0.55 (0.41 – 0.69) | 1.85 (0.29 – 11.65) | n.s. (0.50) / n.s. (0.63) |
| MR-proADM | 0.12 (0.03 – 0.49) | 0.003 | 7.70 (0.006) | 0.66 (0.52 – 0.80) | 0.16 (0.03 – 1.01) | n.s. (0.06) / n.s. (0.15) |
| Bio-ADM | 0.69 (0.28 – 1.70) | n.s. (0.41) | 0.63 (0.43) | 0.54 (0.40 – 0.67) | 0.77 (0.26 – 2.27) | n.s. (0.63) / n.s. (0.63) |
| ADM-Ratio | 1.93 (0.58 – 6.49) | n.s. (0.29) | 1.18 (0.28) | 0.60 (0.45 – 0.74) | 1.56 (0.46 – 5.27) | n.s. (0.47) / NA |
| PAM-AMA x ADM-Ratio interaction | 1.66 (0.62 – 4.50) | n.s. (0.32) | 1.02 (0.32) | 0.58 (0.44 – 0.73) | 1.61 (0.61 – 4.30) | n.s. (0.33) / NA |
| **Incident Vascular Dementia (n= 942/ n=15)** | | | | | | |
| PAM-AMA | 0.66 (0.07 – 6.13) | n.s. (0.72) | 0.13 (0.72) | 0.58 (0.43 – 0.73) | 1.05 (0.13 – 8.22) | n.s. (0.96) / n.s. (0.96) |
| MR-proADM | 0.28 (0.05 – 1.60) | n.s. (0.15) | 1.93 (0.17) | 0.62 (0.47 – 0.77) | 0.80 (0.10 – 6.47) | n.s. (0.83) / n.s. (0.96) |
| Bio-ADM | 0.60 (0.21 – 1.67) | n.s. (0.33) | 0.87 (0.35) | 0.57 (0.37 – 0.77) | 0.67 (0.20 – 2.20) | n.s. (0.51) / n.s. (0.96) |
| ADM-Ratio | 0.93 (0.25 – 3.44) | n.s. (0.92) | 0.01 (0.92) | 0.49 (0.31 – 0.67) | 0.66 (0.18 – 2.50) | n.s. (0.54) / NA |
| PAM-AMA x ADM-Ratio interaction | 0.85 (0.28 – 2.60) | n.s. (0.78) | 0.08 (0.78) | 0.54 (0.36 – 0.72) | 0.77 (0.26 – 2.32) | n.s. (0.65) / NA |

Bio-ADM: bioactive Adrenomedullin, MR-proADM: mid-regional pro-Adrenomedullin, ADM-Ratio: molar ratio of bio-ADM/MR-proADM, PAM-AMA: peptidylglycine-alpha amidating monooxygenase amidating activity. BMI: Body-Mass Index, HR: hazard ratio, CI: confidence interval, C-Index: concordance index, LR-Chi²: Likelihood-Ratio Chi², n.s.: not significant. The HRs per 1 SD are shown with 95% CI. The HRs are presented either in an unadjusted form (Analysis 1) or after adjustment for age, sex, SBP, AHT, smoking, HDL, and prior history of cardiometabolic disease (Analysis 2). The data is presented for the BMI≥30 subgroup. In Analysis 2, p-values for the co-primary markers PAM-AMA, MR-proADM and bio-ADM were corrected for multiple testing using the Benjamini-Hochberg false discovery rate (FDR) method, applied separately for each endpoint and model. Resulting FDR adjusted p-values are shown as q-values. ADM-Ratio and PAM-AMA x ADM-Ratio interaction were considered exploratory and were not included in the FDR correction (q-value shown as NA (not applicable)).

Supplementary Table S4A: Associations between biomarkers (bio-ADM, MR-proADM, ADM-Ratio, PAM-AMA) and dementia risk after exclusion of cases occurring within the first two years of follow-up: Univariable and multivariable-adjusted Cox regression analysis.

|  | **Analysis 1: univariable cox proportional regression** | | | | **Analysis 2: multivariable-adjusted cox proportional regression** | | **Analysis 3: multivariable-adjusted cox proportional regression with BMI as additional covariate** | |
| --- | --- | --- | --- | --- | --- | --- | --- | --- |
| Biomarkers | Unadjusted HR (95%CI) | p value | LR-Chi²  (LR-p value) | C-Index (95% CI) | adjusted HR (95%CI) | p value / q value | adjusted HR (95%CI) | p value / q value |
| **Type of dementia (population size / number of events)** | | | | | | | | |
| **Incident Alzheimer‘s Dementia (n=4543 / n=158)** | | | | | | | | |
| PAM-AMA | 1.97 (1.03 – 3.75) | 0.04 | 4.13 (0.04) | 0.55 (0.50 – 0.60) | 2.01 (1.06 – 3.82) | 0.03 / n.s (0.08) | 1.83 (0.97 – 3.43) | n.s. (0.06) / n.s. (0.19) |
| MR-proADM | 0.66 (0.36 – 1.20) | n.s. (0.17) | 1.82 (0.18) | 0.54 (0.50 – 0.59) | 1.10 (0.55 – 2.23) | n.s. (0.78) n.s. (0.78) | 0.69 (0.33 – 1.43) | n.s. (0.31) / n.s. (0.47) |
| bio-ADM | 1.48 (1.09 – 2.16) | 0.04 | 4.22 (0.04) | 0.56 (0.51 – 0.61) | 1.50 (1.00 – 2.25) | 0.05 / n.s. (0.08) | 1.12 (0.74 – 1.70) | n.s. (0.59) / n.s. (0.60) |
| ADM-ratio | 2.08 (1.32 – 3.25) | 0.001 | 12.81 (0.001) | 0.60 (0.55 – 0.65) | 1.59 (1.01 – 2.50) | 0.04 / N/A | 1.30 (0.83 – 2.04) | n.s. (0.26) / NA |
| PAM-AMA x ADM-ratio interaction | 2.04 (1.41 – 2.958) | 0.0002 | 14.84 (0.0001) | 0.61 (0.56 – 0.66) | 1.71 (1.18 – 2.49) | 0.005 / NA | 1.46 (1.00 – 2.13) | 0.05 / NA |
| **Incident Vascular Dementia (n= 4543/ n=76)** | | | | | | | | |
| PAM-AMA | 0.85 (0.33 – 2.17) | n.s. (0.73) | 0.12 (0.73) | 0.52 (0.45 – 0.59) | 1.09 (0.43 – 2.75) | n.s. (0.85) / n.s. (0.85) | 1.05 (0.42 – 2.63) | n.s. (0.21) / n.s. (0.92) |
| MR-proADM | 0.20 (0.10 – 0.44) | <0.0001 | 14.50 (0.0001) | 0.61 (0.54 – 0.67) | 0.73 (0.28 – 1.91) | n.s. (0.52) / n.s. (0.77) | 0.53 (0.20 – 1.42) | n.s. (0.17) / n.s. (0.45) |
| bio-ADM | 1.23 (0.72 – 2.08) | n.s. (0.44) | 0.60 (0.44) | 0.53 (0.46 – 0.61) | 1.70 (0.95 – 3.03) | n.s. (0.07) / n.s. (0.22) | 1.38 (0.75 – 2.54) | n.s. (0.30) / n.s. (0.45) |
| ADM-ratio | 3.34 (1.70 – 6.54) | 0.0005 | 13.30 (0.0003) | 0.59 (0.52 – 0.65) | 2.18 (1.15 – 4.13) | 0.02 / NA | 1.86 (0.96 – 3.60) | n.s. (0.06) / NA |
| PAM-AMA x ADM-ratio interaction | 2.12 (1.24 – 3.05) | 0.006 | 7.95 (0.005) | 0.57 (0.50 – 0.64) | 1.04 (0.95 – 2.97) | 0.04 / NA | 1.55 (0.91 – 2.65) | n.s. (0.11) / NA |

Bio-ADM: bioactive Adrenomedullin, MR-proADM: mid-regional pro-Adrenomedullin, ADM-Ratio: molar ratio of bio-ADM/MR-proADM, PAM-AMA: peptidylglycine-alpha amidating monooxygenase amidating activity. HR: hazard ratio, CI: confidence interval. C-Index: concordance index, LR-Chi²: Likelihood-Ratio Chi², n.s.: not significant. The HRs per 1 SD are shown with 95% CI. The HRs are presented either in an unadjusted form (Analysis 1) or after adjustment for age, sex, SBP, AHT, smoking, HDL, and prior history of cardiometabolic disease (Analysis 2). Analysis 3 equals to analysis 2 after inclusion of BMI as a covariate. In Analyses 2 and 3, p-values for the co-primary markers PAM-AMA, MR-proADM and bio-ADM were corrected for multiple testing using the Benjamini-Hochberg false discovery rate (FDR) method, applied separately for each endpoint and model. Resulting FDR adjusted p-values are shown as q-values. ADM-Ratio and PAM-AMA x ADM-Ratio interaction were considered exploratory and were not included in the FDR correction (q-value shown as NA (not applicable)).

Supplementary table S4B: Associations between biomarkers (bio-ADM, MR-proADM, ADM-Ratio, PAM-AMA) and dementia risk and dementia risk after exclusion of cases occurring within the first two years of follow-up: Univariable and multivariable-adjusted Cox regression analysis after stratification by BMI (BMI<30).

|  | **Analysis 1 in BMI<30: univariable cox proportional regression** | | | | **Analysis 2 in BMI<30: multivariable-adjusted cox proportional regression** | |
| --- | --- | --- | --- | --- | --- | --- |
| Biomarkers | Unadjusted HR (95%CI) | p value | LR Chi²  (LR-p value) | C-Index (95% CI) | adjusted HR (95%CI) | p value / q value |
| **Type of dementia (population size / number of events)** | | | | | | |
| **Incident Alzheimer‘s Dementia (n=3601 / n=141)** | | | | | | |
| PAM-AMA | 2.16 (1.10 – 4.23) | 0.03 | 4.49 (0.03) | 0.55 (0.50 – 0.60) | 2.08 (1.07 – 4.05) | 0.03 / n.s. (0.09) |
| MR-proADM | 0.66 (0.34 – 1.30) | n.s. (0.23) | 1.41 (0.24) | 0.55 (0.50 – 0.60) | 1.20 (0.55 – 2.66) | n.s. (0.65) / n.s. (0.65) |
| Bio-ADM | 1.38 (0.90 – 2.10) | n.s. (0.14) | 2.20 (0.13) | 0.55 (0.50 – 0.60) | 1.46 (0.93 – 2.27) | n.s. (0.09) / n.s. (0.15) |
| ADM-Ratio | 1.80 (1.11 – 2.91) | 0.02 | 6.07 (0.014) | 0.58 (0.53 – 0.63) | 1.45 (0.90 – 2.33) | n.s. (0.13) / NA |
| PAM-AMA x ADM-Ratio interaction | 1.93 (1.29 – 2.88) | 0.001 | 10.82 (0.001) | 0.60 (0.55 – 0.65) | 1.63 (1.10 – 2.43) | 0.02 / NA |
| **Incident Vascular Dementia (n= 3601/ n=66)** | | | | | | |
| PAM-AMA | 1.00 (0.37 – 2.69) | n.s. (0.99) | <0.001 (0.99) | 0.51 (0.43 – 0.59) | 1.23 (0.46 – 3.29) | n.s. (0.67) / n.s. (0.67) |
| MR-proADM | 0.15 (0.07 – 0.33) | <0.0001 | 17.57 (<0.0001) | 0.62 (0.55 – 0.69) | 0.57 (0.20 – 1.68) | n.s. (0.32) / n.s. (0.47) |
| Bio-ADM | 1.08 (0.60 – 1.93) | n.s. (0.80) | 0.06 (0.80) | 0.52 (0.44 – 0.59) | 1.56 (0.82 – 2.98) | n.s. (0.18) / n.s. (0.47) |
| ADM-Ratio | 3.28 (1.55 – 6.92) | 0.002 | 10.57 (0.001) | 0.60 (0.52 – 0.68) | 2.09 (1.04 – 4.22) | 0.04 / NA |
| PAM-AMA x ADM-Ratio interaction | 2.18 (1.20 – 3.95) | 0.01 | 7.05 (0.008) | 0.57 (0.49 – 0.65) | 1.77 (1.00 – 3.16) | n.s. (0.051) / NA |

Bio-ADM: bioactive Adrenomedullin, MR-proADM: mid-regional pro-Adrenomedullin, ADM-Ratio: molar ratio of bio-ADM/MR-proADM, PAM-AMA: peptidylglycine-alpha amidating monooxygenase amidating activity. BMI: Body-Mass Index, HR: hazard ratio, CI: confidence interval, C-Index: concordance index, LR-Chi²: Likelihood-Ratio Chi², n.s.: not significant. The HRs per 1 SD are shown with 95% CI. The HRs are presented either in an unadjusted form (Analysis 1) or after adjustment for age, sex, SBP, AHT, smoking, HDL, and prior history of cardiometabolic disease (Analysis 2). The data is presented for the BMI<30 subgroup. In Analysis 2, p-values for the co-primary markers PAM-AMA, MR-proADM and bio-ADM were corrected for multiple testing using the Benjamini-Hochberg false discovery rate (FDR) method, applied separately for each endpoint and model. Resulting FDR adjusted p-values are shown as q-values. ADM-Ratio and PAM-AMA x ADM-Ratio interaction were considered exploratory and were not included in the FDR correction (q-value shown as NA (not applicable)).

Supplementary table S4C: Associations between biomarkers (bio-ADM, MR-proADM, ADM-Ratio, PAM-AMA) and dementia risk after exclusion of cases occurring within the first two years of follow-up: Univariable and multivariable-adjusted Cox regression analysis after stratification by BMI (BMI≥30).

|  | **Analysis 1 in BMI**≥**30: univariable cox proportional regression** | | | | **Analysis 2 in BMI**≥**30: multivariable-adjusted cox proportional regression** | |
| --- | --- | --- | --- | --- | --- | --- |
| Biomarkers | Unadjusted HR (95%CI) | p value | LR Chi²  (LR-p value) | C-Index (95% CI) | adjusted HR (95%CI) | p value / q value |
| **Incident Alzheimer‘s Dementia (n=942 / n=17)** | | | | | | |
| PAM-AMA | 0.60 (0.07 – 4.91) | n.s. (0.63) | 0.23 (0.63) | 0.52 (0.35 – 0.69) | 1.00 (0.13 – 7.72) | n.s. (0.99) / n.s. (0.99) |
| MR-proADM | 0.09 (0.02 – 0.44) | 0.003 | 7.93 (0.005) | 0.67 (0.52 – 0.81) | 0.11 (0.02 – 0.78) | 0.03 / n.s. (0.08) |
| Bio-ADM | 0.68 (0.25 – 1.85) | n.s. (0.45) | 0.54 (0.46) | 0.53 (0.47 – 0.80) | 0.78 (0.23 – 2.58) | n.s. (0.67) / n.s. (0.99) |
| ADM-Ratio | 2.19 (0.57 – 8.51) | n.s. (0.26) | 1.34 (0.25) | 0.64 (0.45 – 0.74) | 1.95 (0.49 – 7.73) | n.s. (0.34) / NA |
| PAM-AMA x ADM-Ratio interaction | 1.51 (0.50 – 4.56) | n.s. (0.47) | 0.54 (0.46) | 0.58 (0.40 – 0.75) | 1.56 (0.52 – 4.66) | n.s. (0.43) / NA |
| **Incident Vascular Dementia (n= 942/ n=10)** | | | | | | |
| PAM-AMA | 0.23 (0.02 – 3.23) | n.s. (0.28) | 1.15 (0.28) | 0.62 (0.50 – 0.74) | 0.39 (0.03 – 4.72) | n.s. (0.46) / n.s. (0.80) |
| MR-proADM | 0.30 (0.03 – 2.72) | n.s. (0.28) | 1.10 (0.29) | 0.60 (0.40 – 0.79) | 0.71 (0.05 – 9.45) | n.s. (0.79) / n.s. (0.80) |
| Bio-ADM | 1.19 (0.27 – 5.17) | n.s. (0.82) | 0.05 (0.82) | 0.52 (0.27 – 0.76) | 1.57 (0.32 – 7.54) | n.s. (0.58) / n.s. (0.80) |
| ADM-Ratio | 2.49 (0.42 – 14.6) | n.s. (0.31) | 1.07 (0.30) | 0.62 (0.43 – 0.81) | 2.09 (0.33 – 13.0) | n.s. (0.43) / NA |
| PAM-AMA x ADM-Ratio interaction | 1.25 (0.30 – 5.21) | n.s. (0.76) | 0.10 (0.76) | 0.55 (0.36 – 0.74) | 1.19 (0.29 – 4.77) | n.s. (0.81) / NA |

Bio-ADM: bioactive Adrenomedullin, MR-proADM: mid-regional pro-Adrenomedullin, ADM-Ratio: molar ratio of bio-ADM/MR-proADM, PAM-AMA: peptidylglycine-alpha amidating monooxygenase amidating activity. BMI: Body-Mass Index, HR: hazard ratio, CI: confidence interval, C-Index: concordance index, LR-Chi²: Likelihood-Ratio Chi², n.s.: not significant. The HRs per 1 SD are shown with 95% CI. The HRs are presented either in an unadjusted form (Analysis 1) or after adjustment for age, sex, SBP, AHT, smoking, HDL, and prior history of cardiometabolic disease (Analysis 2). The data is presented for the BMI≥30 subgroup. In Analysis 2, p-values for the co-primary markers PAM-AMA, MR-proADM and bio-ADM were corrected for multiple testing using the Benjamini-Hochberg false discovery rate (FDR) method, applied separately for each endpoint and model. Resulting FDR adjusted p-values are shown as q-values. ADM-Ratio and PAM-AMA x ADM-Ratio interaction were considered exploratory and were not included in the FDR correction (q-value shown as NA (not applicable)).

Supplementary table S4D: Associations between biomarkers (bio-ADM, MR-proADM, ADM-Ratio, PAM-AMA) and dementia risk after exclusion of incident VD cases: Univariable and multivariable-adjusted Cox regression analysis in the full cohort and after stratification by BMI (BMI<30).

|  | **Analysis 1: univariable cox proportional regression** | | | **Analysis 2: multivariable-adjusted cox proportional regression** | |
| --- | --- | --- | --- | --- | --- |
| Biomarkers | Unadjusted HR (95%CI) | p value | LR Chi²  (LR-p value) | adjusted HR (95%CI) | p value |
| **Incident Alzheimer‘s Dementia, full cohort (n=4450 / n=191)** | | | | | |
| PAM-AMA | 2.45 (1.36 – 4.42) | 0.003 | 8.8 (0.0003) | 2.55 (1.43 – 4.54) | 0.002 |
| MR-proADM | 0.81 (0.46 – 1.40) | n.s. (0.44) | 0.57 (0.45) | 1.67 (0.89 – 3.15) | n.s. (0.11) |
| Bio-ADM | 1.56 (1.10 – 2.21) | 0.01 | 6.55 (0.01) | 1.67 (1.14 – 2.43) | 0.008 |
| ADM-Ratio | 2.02 (1.34 – 3.04) | 0.0008 | 12.05 (0.0001) | 1.52 (1.01 – 2.29) | 0.045 |
| PAM-AMA x ADM-Ratio interaction | 2.16 (1.53 – 3.03) | 0.00001 | 20.52 (<0.0001) | 1.81 (1.28 – 2.55) | 0.0007 |
| **Incident Alzheimer‘s Dementia, BMI < 30 (n=3523 / n=172)** | | | | | |
| PAM-AMA | 2.46 (1.33 – 4.54) | 0.004 | 8.2 (0.004) | 2.40 (1.32 – 4.38) | 0.004 |
| MR-proADM | 0.78 (0.42 – 1.45) | n.s. (0.43) | 0.61 (0.44) | 1.83 (0.91 – 3.70) | n.s. (0.09) |
| Bio-ADM | 1.45 (0.98 – 2.13) | n.s. (0.06) | 3.73 (0.05) | 1.64 (1.09 – 2.47) | 0.02 |
| ADM-Ratio | 1.77 (1.15 – 2.75) | 0.01 | 7.07 (0.007) | 1.41 (0.92 – 2.17) | n.s. (0.12) |
| PAM-AMA x ADM-Ratio interaction | 2.00 (1.39 – 2.89) | 0.0002 | 14.58 (0.0001) | 1.69 (1.18 – 2.43) | 0.005 |

Bio-ADM: bioactive Adrenomedullin, MR-proADM: mid-regional pro-Adrenomedullin, ADM-Ratio: molar ratio of bio-ADM/MR-proADM, PAM-AMA: peptidylglycine-alpha amidating monooxygenase amidating activity. BMI: Body-Mass Index, HR: hazard ratio, CI: confidence interval, LR-Chi²: Likelihood-Ratio Chi², n.s.: not significant. The HRs per 1 SD are shown with 95% CI. The HRs are presented either in an unadjusted form (Analysis 1) or after adjustment for age, sex, SBP, AHT, smoking, HDL, and prior history of cardiometabolic disease (Analysis 2). The data is presented for the full cohort (top) and the BMI<30 subgroup (bottom).

Supplementary table S4E: Bootstrap based internal validation of continuous incident AD models using the concordance index.

|  | Apparent C-Index | Optimism mean | Optimism corrected C-index | Bootstrap samples (B) |
| --- | --- | --- | --- | --- |
| **Incident Alzheimer‘s Dementia, full cohort (n=4543 / n=205)** | | | | |
| Base Model | 0.2987 | -0.0133 | 0.3120 | 300 |
| Base Model + PAM-AMA | 0.2892 | -0.0149 | 0.3040 | 300 |
| Base Model + Bio-ADM | 0.2903 | -0.0155 | 0.3058 | 300 |
| Base Model + ADM-Ratio | 0.2938 | -0.0151 | 0.3090 | 300 |
| Base Model + PAM-AMA x ADM-Ratio interaction | 0.2857 | -0.0148 | 0.3004 | 300 |
| **Incident Alzheimer‘s Dementia, BMI < 30 (n=3601 / n=184)** | | | | |
| Base Model | 0.3045 | -0.0145 | 0.3190 | 300 |
| Base Model + PAM-AMA | 0.2950 | -0.0164 | 0.3114 | 300 |
| Base Model + Bio-ADM | 0.2985 | -0.0175 | 0.3160 | 300 |
| Base Model + ADM-Ratio | 0.3018 | -0.0173 | 0.3191 | 300 |
| Base Model + PAM-AMA x ADM-Ratio interaction | 0.2952 | -0.0173 | 0.3125 | 300 |

Bio-ADM: bioactive Adrenomedullin, ADM-Ratio: molar ratio of bio-ADM/MR-proADM, PAM-AMA: peptidylglycine-alpha amidating monooxygenase amidating activity. BMI: Body-Mass Index, B: number of bootstrap resamples. C-Index: concordance index, reported on the scale returned by the bootstrap concordance procedure and are therefore not directly comparable to Harrell’s C-Index values commonly reported on a 0.5–1.0 scale. Optimism was defined as the mean difference between model discrimination in bootstrap samples and performance in the original dataset. Optimism corrected C-Indices were obtained by subtracting the estimated optimism mean from the apparent C-Index. The base model is constructed of age, sex, SBP, AHT, smoking, HDL, and prior history of cardiometabolic disease. The data is presented for the full cohort (top) and the BMI<30 subgroup (bottom).

Supplementary table S5: Categorical Associations between biomarkers (bio-ADM, MR-proADM, ADM-Ratio, PAM-AMA) and dementia risk after stratification by quartiles: Univariable Cox regression in the full sample cohort.

|  |  | **Incident Alzheimer Dementia** | | **Incident Vascular Dementia** | |
| --- | --- | --- | --- | --- | --- |
|  | **n** |  |  |  |  |
| **Biomarker quartiles** |  | **HR (95% CI)** | **p-value** | **HR (95% CI)** | **p-value** |
| **PAM-AMA** | | | | | |
| p-value linear trend (LR-Chi² / LR-p value) | 0.02 (5.43 / 0.14) | | | 0.94 (0.23 / 0.97) | |
| Q1 (3.8 – 10.8 Units) | 1136 | 1.56 (1.05 – 2.30) | 0.027 | 1.02 (0.58 – 1.78) | n.s. (0.95) |
| Q2 (10.8 – 12.5 Units) | 1136 | 1.33 (0.89 – 1.99) | n.s. (0.16) | 0.92 (0.52 – 1.64) | n.s. (0.78) |
| Q3 (12.5 – 14.5 Units) | 1135 | 1.17 (0.78 – 1.77) | n.s. (0.45) | 0.91 (0.51 – 1.61) | n.s. (0.74) |
| Q4 (14.5 – 72.2 Units) | 1136 | Reference | | Reference | |
| **MR-proADM** | | | | | |
| p-value linear trend (LR-Chi² / LR-p value) | 0.216 (4.83 / 0.19) | | | <0.0001 (22.90 / <0.0001) | |
| Q1 (0.121 – 0.609 nM) | 1136 | 0.75 (0.51 – 1.10) | n.s. (0.15) | 0.22 (0.10 – 0.47) | <0.0001 |
| Q2 (0.609 – 0.705 nM) | 1136 | 0.79 (0.55 – 1.14) | n.s. (0.21) | 0.51 (0.30 – 0.88) | 0.02 |
| Q3 (0.705 – 0.837 nM) | 1135 | 0.68 (0.47 – 1.00) | 0.05 | 0.66 (0.41 – 1.10) | n.s. (0.10) |
| Q4 (0.838 – 4.376 nM) | 1136 | Reference | | Reference | |
| **bio-ADM** | | | | | |
| p-value linear trend (LR-Chi² / LR-p value) | 0.02 (7.34 / 0.06) | | | 0.39 (4.69 / 0.20) | |
| Q1 (8 – 10.4 pg/mL) | 1136 | 1.62 (1.10 – 2.37) | 0.014 | 1.09 (0.63 – 1.88) | n.s. (0.77) |
| Q2 (10.4 – 14.0 pg/mL) | 1136 | 1.10 (0.72 – 1.64) | n.s. (0.71) | 1.11 (0.64 – 1.90) | n.s. (0.72) |
| Q3 (14.0 – 18.9 pg/mL) | 1136 | 1.16 (0.77 – 1.75) | n.s. (0.47) | 0.60 (0.32 – 1.15) | n.s. (0.12) |
| Q4 (18.9 – 283 pg/mL) | 1135 | Reference | | Reference | |
| **ADM-Ratio (x10^-^³)** | | | | | |
| p-value linear trend (LR-Chi² / LR-p value) | <0.0001 (16.07 / 0.001) | | | 0.002 (13.12 / 0.004) | |
| Q1 (0.752 – 2.57) | 1136 | 2.12 (1.42 – 3.18) | 0.0001 | 2.04 (1.16 – 3.59) | 0.014 |
| Q2 (2.57 – 3.22) | 1136 | 1.60 (1.04 – 2.45) | 0.03 | 1.50 (0.82 – 2.73) | n.s. (0.19) |
| Q3 (3.23 – 4.10) | 1135 | 1.26 (0.81 – 1.97) | n.s. (0.31) | 0.74 (0.36 – 1.51) | n.s. (0.41) |
| Q4 (4.10 – 98.4) | 1136 | Reference | | Reference | |
| **Interaction-Term ADM-Ratio x PAM-AMA (x10^-3^)** | | | | | |
| p-value linear trend (LR-Chi² / LR-p value) | <0.0001 (24.48 / <0.0001) | | | 0.012 (11.32 / 0.01) | |
| Q1 (6.5 – 30.7 Units) | 1136 | 2.64 (1.74 – 4.00) | <0.0001 | 1.68 (0.96 – 2.94) | n.s. (0.07) |
| Q2 (30.7 – 40.0 Units) | 1136 | 1.81 (1.16 – 2.81) | 0.009 | 1.44 (0.81 – 2.55) | n.s. (0.22) |
| Q3 (40.0 – 53.8 Units) | 1135 | 1.46 (0.92 – 2.31) | n.s. (0.11) | 0.66 (0.33 – 1.32) | n.s. (0.24) |
| Q4 (53.9 – 1989 Units) | 1136 | Reference | | Reference | |

Bio-ADM: bioactive Adrenomedullin, MR-proADM: mid-regional pro-Adrenomedullin, ADM-Ratio: molar ratio of bio-ADM/MR-proADM, PAM-AMA: peptidylglycine-alpha amidating monooxygenase amidating activity. BMI: Body-Mass Index, HR: hazard ratio, CI: confidence interval, LR-Chi²: Likelihood-Ratio Chi², n.s.: not significant. Q1-Q4: Quartiles. The HRs are shown with 95% CI, Q4 was the reference quartile in all analyses.

Supplementary table S6: Categorical associations between biomarkers (bio-ADM, MR-proADM, ADM-Ratio, PAM-AMA) and dementia risk after stratification by quartiles: Multivariable-adjusted Cox regression in the full sample cohort.

|  |  | **Incident All-cause Dementia (AD+VD)** | | **Incident Alzheimer Dementia** | |
| --- | --- | --- | --- | --- | --- |
|  | **n** |  |  |  |  |
| **Biomarker quartiles** |  | **aHR (95% CI)** | **p-value** | **aHR (95% CI)** | **p-value** |
| **PAM-AMA** | | | | | |
| p-value linear trend | 0.01 | | | 0.47 | |
| Q1 (3.8 – 10.8 Units) | 1136 | 1.64 (1.10 – 2.42) | 0.01 | 1.20 (0.68 – 2.10) | n.s. (0.53) |
| Q2 (10.8 – 12.5 Units) | 1136 | 1.37 (0.92 – 2.06) | n.s. (0.12) | 1.11 (0.62 – 1.97) | n.s. (0.74) |
| Q3 (12.5 – 14.5 Units) | 1135 | 1.20 (0.79 – 1.81) | n.s. (0.39) | 0.97 (0.55 – 1.72) | n.s. (0.92) |
| Q4 (14.5 – 72.2 Units) | 1136 | Reference | | Reference | |
| **MR-proADM** | | | | | |
| p-value linear trend | 0.26 | | | 0.53 | |
| Q1 (0.121 – 0.609 nM) | 1136 | 1.22 (0.79 – 1.88) | n.s. (0.37) | 0.65 (0.28 – 1.48) | n.s. (0.30) |
| Q2 (0.609 – 0.705 nM) | 1136 | 1.09 (0.73 – 1.63) | n.s. (0.67) | 1.09 (0.61 – 1.97) | n.s. (0.77) |
| Q3 (0.705 – 0.837 nM) | 1135 | 0.84 (0.57 – 1.25) | n.s. (0.39) | 1.03 (0.62 – 1.72) | n.s. (0.91) |
| Q4 (0.838 – 4.376 nM) | 1136 | Reference | | Reference | |
| **Bio-ADM** | | | | | |
| p-value linear trend | 0.01 | | | 0.03 | |
| Q1 (8 – 10.4 pg/mL) | 1136 | 1.73 (1.15 – 2.59) | 0.009 | 1.69 (0.95 – 3.03) | n.s. (0.08) |
| Q2 (10.4 – 14.0 pg/mL) | 1136 | 1.15 (0.75 – 1.76) | n.s. (0.53) | 1.40 (0.80 – 2.45) | n.s. (0.24) |
| Q3 (14.0 – 18.9 pg/mL) | 1136 | 1.18 (0.79 – 1.79) | n.s. (0.42) | 0.71 (0.37 – 1.35) | n.s. (0.29) |
| Q4 (18.9 – 283 pg/mL) | 1135 | Reference | | Reference | |
| **ADM-Ratio (x10^-^³)** | | | | | |
| p-value linear trend | 0.01 | | | 0.05 | |
| Q1 (0.752 – 2.57) | 1136 | 1.64 (1.08 – 2.50) | 0.02 | 1.44 (0.80 – 2.60) | n.s. (0.22) |
| Q2 (2.57 – 3.22) | 1136 | 1.39 (0.90 – 2.14) | n.s. (0.14) | 1.34 (0.70 – 2.47) | n.s. (0.35) |
| Q3 (3.23 – 4.10) | 1135 | 1.12 (0.71 – 1.76) | n.s. (0.62) | 0.63 (0.29 – 1.28) | n.s. (0.20) |
| Q4 (4.10 – 98.4) | 1136 | Reference | | Reference | |
| **Interaction-Term ADM-Ratio x PAM-AMA (x10^-3^)** | | | | | |
| p-value linear trend | <0.0001 | | | 0.07 | |
| Q1 (6.5 – 30.7 Units) | 1136 | 2.20 (1.43 – 3.37) | <0.0001 | 1.43 (0.81 – 2.54) | n.s. (0.22) |
| Q2 (30.7 – 40.0 Units) | 1136 | 1.65 (1.06 – 2.59) | 0.03 | 1.36 (0.76 – 2.44) | n.s. (0.30) |
| Q3 (40.0 – 53.8 Units) | 1135 | 1.41 (0.89 – 2.24) | n.s. (0.15) | 0.68 (0.34 – 1.36) | n.s. (0.27) |
| Q4 (53.9 – 1989 Units) | 1136 | Reference | | Reference | |

Bio-ADM: bioactive Adrenomedullin, MR-proADM: mid-regional pro-Adrenomedullin, ADM-Ratio: molar ratio of bio-ADM/MR-proADM, PAM-AMA: peptidylglycine-alpha amidating monooxygenase amidating activity. BMI: Body-Mass Index, HR: hazard ratio, CI: confidence interval, LR-Chi²: Likelihood-Ratio Chi², n.s.: not significant. Q1-Q4: Quartiles. The adjusted HRs are shown with 95% CI, after adjustment for age, sex, SBP, AHT, smoking, HDL, and prior history of cardiometabolic disease. Q4 was the reference quartile in all analyses.

Supplementary table S7: Categorical associations between biomarkers (bio-ADM, MR-proADM, ADM-Ratio, PAM-AMA) and dementia risk after stratification by quartiles: Multivariable-adjusted Cox regression including BMI as covariable in the full sample cohort.

|  |  | **Incident All-cause Dementia (AD+VD)** | | **Incident Alzheimer Dementia** | |
| --- | --- | --- | --- | --- | --- |
|  | **n** |  |  |  |  |
| **Biomarker quartiles** |  | **aHR (95% CI)** | **p-value** | **aHR (95% CI)** | **p-value** |
| **PAM-AMA** | | | | | |
| p-value linear trend | 0.02 | | | 0.51 | |
| Q1 (3.8 – 10.8 Units) | 1136 | 1.55 (1.04 – 2.30) | 0.03 | 1.18 (0.67 – 2.07) | n.s. (0.57) |
| Q2 (10.8 – 12.5 Units) | 1136 | 1.35 (0.90 – 2.02) | n.s. (0.15) | 1.09 (0.61 – 1.94) | n.s. (0.78) |
| Q3 (12.5 – 14.5 Units) | 1135 | 1.17 (0.78 – 1.77) | n.s. (0.45) | 0.95 (0.54 – 1.69) | n.s. (0.86) |
| Q4 (14.5 – 72.2 Units) | 1136 | Reference | | Reference | |
| **MR-proADM** | | | | | |
| p-value linear trend | 0.67 | | | 0.27 | |
| Q1 (0.121 – 0.609 nM) | 1136 | 0.86 (0.55 – 1.35) | n.s. (0.51) | 0.54 (0.23 – 1.25) | n.s. (0.15) |
| Q2 (0.609 – 0.705 nM) | 1136 | 0.88 (0.58 – 1.32) | n.s. (0.53) | 0.97 (0.53 – 1.77) | n.s. (0.92) |
| Q3 (0.705 – 0.837 nM) | 1135 | 0.74 (0.50 – 1.10) | n.s. (0.14) | 0.95 (0.57 – 1.59) | n.s. (0.85) |
| Q4 (0.838 – 4.376 nM) | 1136 | Reference | | Reference | |
| **Bio-ADM** | | | | | |
| p-value linear trend | 0.59 | | | 0.10 | |
| Q1 (8 – 10.4 pg/mL) | 1136 | 1.17 (0.76 – 1.80) | n.s. (0.48) | 1.47 (0.79 – 2.74) | n.s. (0.23) |
| Q2 (10.4 – 14.0 pg/mL) | 1136 | 0.89 (0.58 – 1.37) | n.s. (0.60) | 1.27 (0.71 – 2.26) | n.s. (0.42) |
| Q3 (14.0 – 18.9 pg/mL) | 1136 | 1.04 (0.69 – 1.57) | n.s. (0.86) | 0.67 (0.35 – 1.28) | n.s. (0.22) |
| Q4 (18.9 – 283 pg/mL) | 1135 | Reference | | Reference | |
| **ADM-Ratio (x10^-^³)** | | | | | |
| p-value linear trend | 0.22 | | | 0.12 | |
| Q1 (0.752 – 2.57) | 1136 | 1.27 (0.83 – 1.96) | n.s. (0.27) | 1.29 (0.70 – 2.37) | n.s. (0.42) |
| Q2 (2.57 – 3.22) | 1136 | 1.18 (0.76 – 1.83) | n.s. (0.46) | 1.25 (0.67 – 2.32) | n.s. (0.48) |
| Q3 (3.23 – 4.10) | 1135 | 1.05 (0.67 – 1.65) | n.s. (0.83) | 0.61 (0.30 – 1.24) | n.s. (0.17) |
| Q4 (4.10 – 98.4) | 1136 | Reference | | Reference | |
| **Interaction-Term ADM-Ratio x PAM-AMA (x10^-3^)** | | | | | |
| p-value linear trend | 0.01 | | | 0.15 | |
| Q1 (6.5 – 30.7 Units) | 1136 | 1.76 (1.14 – 2.72) | 0.011 | 1.28 (0.71 – 2.31) | n.s. (0.41) |
| Q2 (30.7 – 40.0 Units) | 1136 | 1.41 (0.90 – 2.22) | n.s. (0.13) | 1.26 (0.70 – 2.27) | n.s. (0.45) |
| Q3 (40.0 – 53.8 Units) | 1135 | 1.32 (0.83 – 2.09) | n.s. (0.24) | 0.65 (0.32 – 1.30) | n.s. (0.22) |
| Q4 (53.9 – 1989 Units) | 1136 | Reference | | Reference | |

Bio-ADM: bioactive Adrenomedullin, MR-proADM: mid-regional pro-Adrenomedullin, ADM-Ratio: molar ratio of bio-ADM/MR-proADM, PAM-AMA: peptidylglycine-alpha amidating monooxygenase amidating activity. BMI: Body-Mass Index, HR: hazard ratio, CI: confidence interval, n.s.: not significant. Q1-Q4: Quartiles. The adjusted HRs are shown with 95% CI, after adjustment for age, sex, SBP, AHT, smoking, HDL, and prior history of cardiometabolic disease and BMI. Q4 was the reference quartile in all analyses.

Supplementary table S8: Categorical Associations between biomarkers (bio-ADM, MR-proADM, ADM-Ratio, PAM-AMA) and dementia risk after stratification by quartiles: Univariable Cox regression in the BMI≥30 subgroup.

|  |  | **Incident Alzheimer Dementia** | | **Incident Vascular Dementia** | |
| --- | --- | --- | --- | --- | --- |
|  | **n** |  |  |  |  |
| **Biomarker quartiles** |  | **aHR (95% CI)** | **p-value** | **aHR (95% CI)** | **p-value** |
| **PAM-AMA** | | | | | |
| p-value linear trend (LR-Chi² / LR-p value) | 0.80 (0.22 / 0.98) | | | 0.31 (2.23 / 0.53) | |
| Q1 (4.4 – 10.9 Units) | 236 | 1.09 (0.32 – 3.77) | n.s. (0.89) | 0.50 (0.09 – 2.75) | n.s. (0.43) |
| Q2 (10.9 – 12.5 Units) | 235 | 1.28 (0.39 – 4.20) | n.s. (0.68) | 0.75 (0.17 – 3.36) | n.s. (0.71) |
| Q3 (12.5 – 14.5 Units) | 235 | 1.00 (0.29 – 3.48) | n.s. (0.99) | 1.49 (0.42 – 5.27) | n.s. (0.54) |
| Q4 (14.5 – 29.6 Units) | 236 | Reference | | Reference | |
| **MR-proADM** | | | | | |
| p-value linear trend (LR-Chi² / LR-p value) | 0.01 (24.72 / 0.01) | | | 0.16 (2.13 / 0.55) | |
| Q1 (0.259 – 0.673 nM) | 236 | 0.27 (0.07 – 0.98) | 0.05 | 0.35 (0.07 – 1.73) | n.s. (0.20) |
| Q2 (0.673 – 0.782 nM) | 235 | 0.17 (0.04 – 0.78) | 0.02 | 0.50 (0.13 – 2.00) | n.s. (0.33) |
| Q3 (0.783 – 0.952 nM) | 235 | 0.42 (0.15 – 1.21) | n.s. (0.11) | 0.64 (0.18 – 2.27) | n.s. (0.49) |
| Q4 (0.953 – 3.281 nM) | 236 | Reference | | Reference | |
| **Bio-ADM** | | | | | |
| p-value linear trend (LR-Chi² / LR-p value) | 0.22 (5.35 / 0.15) | | | 0.60 (12.05 / 0.007) | |
| Q1 (8 – 14.4 pg/mL) | 236 | 0.60 (0.14 – 2.52) | n.s. (0.49) | 0.88 (0.30 – 2.63) | n.s. (0.83) |
| Q2 (14.4 – 18.5 pg/mL) | 235 | 0.60 (0.14 – 2.53) | n.s. (0.49) | N/A (N/A) | N/A |
| Q3 (18.5 – 24.5 pg/mL) | 235 | 1.94 (0.66 – 5.68) | n.s. (0.23) | 0.28 (0.06 – 1.34) | n.s. (0.11) |
| Q4 (24.6 – 278 pg/mL) | 236 | Reference | | Reference | |
| **ADM-Ratio (x10^-^³)** | | | | | |
| p-value linear trend (LR-Chi² / LR-p value) | 0.61 (2.59 / 0.46) | | | 0.54 (8.75 / 0.03) | |
| Q1 (1.42 – 3.06) | 236 | 1.33 (0.46 – 3.85) | n.s. (0.59) | 1.02 (0.29 – 3.51) | n.s. (0.98) |
| Q2 (3.06 – 3.82) | 235 | 0.53 (0.13 – 2.11) | n.s. (0.36) | 1.04 (0.30 – 2.58) | n.s. (0.96) |
| Q3 (3.83 – 4.78) | 235 | 0.67 (0.19 – 2.36) | n.s. (0.53) | N/A (N/A) | N/A |
| Q4 (4.78 – 45.1) | 236 | Reference | | Reference | |
| **Interaction-Term ADM-Ratio x PAM-AMA (x10^-3^)** | | | | | |
| p-value linear trend (LR-Chi² / LR-p value) | 0.70 (1.49 / 0.69) | | | 0.88 (2.11 / 0.55) | |
| Q1 (6.5 – 36.6 Units) | 236 | 1.23 (0.41 – 3.65) | n.s. (0.72) | 1.03 (0.30 – 3.55) | n.s. (0.97) |
| Q2 (36.6 – 48.1 Units) | 235 | 0.68 (0.19 – 2.39) | n.s. (0.54) | 0.59 (0.14 – 2.46) | n.s. (0.47) |
| Q3 (48.1 – 63.6 Units) | 235 | 0.65 (0.18 – 2.31) | n.s. (0.51) | 0.39 (0.08 – 2.00) | n.s. (0.26) |
| Q4 (63.6 – 700.5 Units) | 236 | Reference | | Reference | |

Bio-ADM: bioactive Adrenomedullin, MR-proADM: mid-regional pro-Adrenomedullin, ADM-Ratio: molar ratio of bio-ADM/MR-proADM, PAM-AMA: peptidylglycine-alpha amidating monooxygenase amidating activity. BMI: Body-Mass Index, HR: hazard ratio, CI: confidence interval, LR-Chi²: Likelihood-Ratio Chi², n.s.: not significant. Q1-Q4: Quartiles. The HRs are shown with 95% CI, Q4 was the reference quartile in all analyses.

Supplementary table S9: Categorical Associations between biomarkers (bio-ADM, MR-proADM, ADM-Ratio, PAM-AMA) and dementia risk after stratification by quartiles: Multivariable-adjusted Cox regression in the BMI≥30 subgroup.

|  |  | **Incident Alzheimer Dementia** | | **Incident Vascular Dementia** | |
| --- | --- | --- | --- | --- | --- |
|  | **n** |  |  |  |  |
| **Biomarker quartiles** |  | **aHR (95% CI)** | **p-value** | **aHR (95% CI)** | **p-value** |
| **PAM-AMA** | | | | | |
| p-value linear trend | 0.48 | | | 0.49 | |
| Q1 (4.4 – 10.9 Units) | 236 | 1.46 (0.41 – 5.17) | n.s. (0.56) | 0.61 (0.11 – 3.38) | n.s. (0.57) |
| Q2 (10.9 – 12.5 Units) | 235 | 1.44 (0.43 – 4.79) | n.s. (0.56) | 1.03 (0.23 – 4.65) | n.s. (0.97) |
| Q3 (12.5 – 14.5 Units) | 235 | 1.09 (0.31 – 3.79) | n.s. (0.90) | 1.84 (0.51 – 6.63) | n.s. (0.35) |
| Q4 (14.5 – 29.6 Units) | 236 | Reference | | Reference | |
| **MR-proADM** | | | | | |
| p-value linear trend | 0.12 | | | 0.89 | |
| Q1 (0.259 – 0.673 nM) | 236 | 0.41 (0.10 – 1.74) | n.s. (0.23) | 0.80 (0.14 – 4.66) | n.s. (0.81) |
| Q2 (0.673 – 0.782 nM) | 235 | 0.28 (0.06 – 1.37) | n.s. (0.16) | 1.14 (0.24 – 5.34) | n.s. (0.87) |
| Q3 (0.783 – 0.952 nM) | 235 | 0.43 (0.14 – 1.29) | n.s. (0.13) | 0.94 (0.26 – 3.49) | n.s. (0.93) |
| Q4 (0.953 – 3.281 nM) | 236 | Reference | | Reference | |
| **Bio-ADM** | | | | | |
| p-value linear trend | 0.47 | | | 0.89 | |
| Q1 (8 – 14.4 pg/mL) | 236 | 0.77 (0.18 – 3.36) | n.s. (0.73) | 1.17 (0.36 – 3.83) | n.s. (0.80) |
| Q2 (14.4 – 18.5 pg/mL) | 235 | 0.78 (0.18 – 3.37) | n.s. (0.74) | N/A (N/A) | N/A |
| Q3 (18.5 – 24.5 pg/mL) | 235 | 2.15 (0.71 – 6.50) | n.s. (0.18) | 0.34 (0.07 – 1.65) | n.s. (0.18) |
| Q4 (24.6 – 278 pg/mL) | 236 | Reference | | Reference | |
| **ADM-Ratio (x10^-^³)** | | | | | |
| p-value linear trend | 0.79 | | | 0.91 | |
| Q1 (1.42 – 3.06) | 236 | 1.16 (0.39 – 3.51) | n.s. (0.79) | 0.78 (0.21 – 2.92) | n.s. (0.71) |
| Q2 (3.06 – 3.82) | 235 | 0.50 (0.12 – 2.05) | n.s. (0.34) | 0.92 (0.26 – 3.29) | n.s. (0.90) |
| Q3 (3.83 – 4.78) | 235 | 0.64 (0.18 – 2.26) | n.s. (0.48) | N/A (N/A) | N/A |
| Q4 (4.78 – 45.1) | 236 | Reference | | Reference | |
| **Interaction-Term ADM-Ratio x PAM-AMA (x10^-3^)** | | | | | |
| p-value linear trend | 0.65 | | | 0.95 | |
| Q1 (6.5 – 36.6 Units) | 236 | 1.23 (0.40 – 3.78) | n.s. (0.71) | 0.87 (0.24 – 3.15) | n.s. (0.83) |
| Q2 (36.6 – 48.1 Units) | 235 | 0.80 (0.22 – 2.94) | n.s. (0.74) | 0.74 (0.17 – 3.16) | n.s. (0.68) |
| Q3 (48.1 – 63.6 Units) | 235 | 0.66 (0.19 – 2.35) | n.s. (0.52) | 0.45 (0.09 – 2.36) | n.s. (0.35) |
| Q4 (63.6 – 700.5 Units) | 236 | Reference | | Reference | |

Bio-ADM: bioactive Adrenomedullin, MR-proADM: mid-regional pro-Adrenomedullin, ADM-Ratio: molar ratio of bio-ADM/MR-proADM, PAM-AMA: peptidylglycine-alpha amidating monooxygenase amidating activity. BMI: Body-Mass Index, HR: hazard ratio, CI: confidence interval, n.s.: not significant. Q1-Q4: Quartiles. The adjusted HRs are shown with 95% CI, after adjustment for age, sex, SBP, AHT, smoking, HDL, and prior history of cardiometabolic disease. Q4 was the reference quartile in all analyses.

Supplementary table S10: Categorical Associations between biomarkers (bio-ADM, MR-proADM, ADM-Ratio, PAM-AMA) and dementia risk after exclusion of cases occurring within the first two years of follow-up and stratification by quartiles: Univariable and multivariable-adjusted Cox regression in the full sample cohort.

|  |  | **Incident Alzheimer Dementia** | | | | **Incident Vascular Dementia** | | | |
| --- | --- | --- | --- | --- | --- | --- | --- | --- | --- |
|  | **n** | Univariable Cox regression | | Multivariable-adjusted Cox regression | | Univariable Cox regression | | Multivariable-adjusted Cox regression | |
| **Biomarker quartiles** |  | **aHR (95% CI)** | **p-value** | **aHR (95% CI)** | **p-value** | **aHR (95% CI)** | **p-value** | **aHR (95% CI)** | **p-value** |
| **PAM-AMA** | | | | | | | | | |
| p-value linear trend (LR-Chi² / LR-p value) | 0.03 (4.89 / 0.18) | | | 0.02 | | 0.94 (0.40 / 0.94) | | 0.58 | |
| Q1 (3.8 – 10.8 Units) | 1136 | 1.59 (1.03 – 2.48) | 0.04 | 1.66 (1.07 – 2.59) | 0.02 | 0.92 (0.49 – 1.73) | n.s. (0.80) | 1.09 (0.58 – 2.06) | n.s. (0.78) |
| Q2 (10.8 – 12.4 Units) | 1136 | 1.31 (0.83 – 2.06) | n.s. (0.25) | 1.34 (0.84 – 2.11) | n.s. (0.22) | 1.01 (0.55 – 1.86) | n.s. (0.98) | 1.22 (0.66 – 2.26) | n.s. (0.53) |
| Q3 (12.4 – 14.5 Units) | 1135 | 1.13 (0.71 – 1.81) | n.s. (0.61) | 1.15 (0.72 – 1.84) | n.s. (0.56) | 0.84 (0.44 – 1.60) | n.s. (0.60) | 0.90 (0.47 – 1.70) | n.s. (0.74) |
| Q4 (14.5 – 72.1 Units) | 1136 | Reference | | Reference | | Reference | | Reference | |
| **MR-proADM** | | | | | | | | | |
| p-value linear trend (LR-Chi² / LR-p value) | 0.18 (3.50 / 0.32) | | | 0.82 | | 0.001 (12.47 / 0.006) | | 0.79 | |
| Q1 (0.121 – 0.609 nM) | 1136 | 0.71 (0.45 – 1.10) | n.s. (0.13) | 1.00 (0.61 – 1.66) | n.s. (0.99) | 0.29 (0.13 – 0.64) | 0.002 | 0.80 (0.34 – 1.89) | n.s. (0.61) |
| Q2 (0.609 – 0.705 nM) | 1136 | 0.80 (0.52 – 1.21) | n.s. (0.29) | 1.01 (0.64 – 1.59) | n.s. (0.98) | 0.54 (0.29 – 0.99) | 0.045 | 1.10 (0.57 – 2.13) | n.s. (0.78) |
| Q3 (0.705 – 0.837 nM) | 1135 | 0.70 (0.46 – 1.07) | n.s. (0.10) | 0.81 (0.52 – 1.26) | n.s. (0.34) | 0.74 (0.43 – 1.27) | n.s. (0.27) | 1.10 (0.62 – 1.94) | n.s. (0.74) |
| Q4 (0.838 – 4.376 nM) | 1136 | Reference | | Reference | | Reference | | Reference | |
| **Bio-ADM** | | | | | | | | | |
| p-value linear trend (LR-Chi² / LR-p value) | 0.06 (7.21 / 0.07) | | | 0.063 | | 0.11 (6.59 / 0.09) | | 0.006 | |
| Q1 (8 – 10.4 pg/mL) | 1136 | 1.59 (1.04 – 2.44) | 0.03 | 1.61 (1.02 – 2.54) | 0.04 | 1.39 (0.77 – 2.53) | n.s. (0.28) | 2.16 (1.14 – 4.07) | 0.02 |
| Q2 (10.4 – 14.0 pg/mL) | 1136 | 0.93 (0.58 – 1.51) | n.s. (0.77) | 0.95 (0.58 – 1.56) | n.s. (0.85) | 1.14 (0.61 – 2.12) | n.s. (0.68) | 1.46 (0.77 – 2.77) | n.s. (0.24) |
| Q3 (14.0 – 18.9 pg/mL) | 1135 | 1.12 (0.71 – 1.76) | n.s. (0.64) | 1.12 (0.70 – 1.77) | n.s. (0.64) | 0.58 (0.28 – 1.23) | n.s. (0.16) | 0.69 (0.33 – 1.45) | n.s. (0.33) |
| Q4 (18.9 – 283 pg/mL) | 1136 | Reference | | Reference | | Reference | | Reference | |
| **ADM-Ratio (x10^-^³)** | | | | | | | | | |
| p-value linear trend (LR-Chi² / LR-p value) | <0.0001 (14.56 / 0.002) | | | 0.011 | | 0.002 (11.94 / 0.008) | | 0.03 | |
| Q1 (0.752 – 2.57) | 1136 | 2.07 (1.32 – 3.26) | 0.002 | 1.63 (1.02 – 2.62) | 0.042 | 2.26 (1.20 – 4.25) | 0.011 | 1.66 (0.86 – 3.22) | n.s. (0.13) |
| Q2 (2.57 – 3.22) | 1136 | 1.64 (1.02 – 2.64) | 0.04 | 1.43 (0.88 – 2.33) | n.s. (0.15) | 1.49 (0.75 – 2.95) | n.s. (0.25) | 1.36 (0.68 – 2.74) | n.s. (0.38) |
| Q3 (3.23 – 4.10) | 1135 | 1.07 (0.64 – 1.79) | n.s. (0.81) | 0.96 (0.57 – 1.62) | n.s. (0.87) | 0.81 (0.37 – 1.78) | n.s. (0.60) | 0.70 (0.32 – 1.54) | n.s. (0.37) |
| Q4 (4.10 – 98.4) | 1136 | Reference | | Reference | | Reference | | Reference | |
| **Interaction-Term ADM-Ratio x PAM-AMA (x10^-3^)** | | | | | | | | | |
| p-value linear trend (LR-Chi² / LR-p value) | <0.0001 (20.62 / <0.0001) | | | <0.0001 | | 0.009 (9.65 / 0.02) | | 0.04 | |
| Q1 (6.5 – 30.7 Units) | 1136 | 2.74 (1.70 – 4.40) | <0.0001 | 2.29 (1.48 – 3.72) | 0.001 | 1.91 (1.01 – 3.58) | 0.045 | 1.68 (0.88 – 3.20) | n.s. (0.12) |
| Q2 (30.7 – 40.0 Units) | 1136 | 1.75 (1.05 – 2.90) | 0.03 | 1.61 (0.97 – 2.69) | n.s. (0.07) | 1.58 (0.83 – 3.03) | n.s. (0.17) | 1.52 (0.78 – 2.93) | n.s. (0.22) |
| Q3 (40.0 – 53.8 Units) | 1135 | 1.46 (0.87 – 2.47) | n.s. (0.16) | 1.42 (0.84 – 2.39) | n.s. (0.20) | 0.74 (0.34 – 1.62) | n.s. (0.45) | 0.77 (0.35 – 1.69) | n.s. (0.52) |
| Q4 (53.9 – 1989 Units) | 1136 | Reference | | Reference | | Reference | | Reference | |

Bio-ADM: bioactive Adrenomedullin, MR-proADM: mid-regional pro-Adrenomedullin, ADM-Ratio: molar ratio of bio-ADM/MR-proADM, PAM-AMA: peptidylglycine-alpha amidating monooxygenase amidating activity. BMI: Body-Mass Index, HR: hazard ratio, CI: confidence interval, C-Index: concordance index, LR-Chi²: Likelihood-Ratio Chi², n.s.: not significant. Q1-Q4: Quartiles. The HRs are shown with 95% CI, Q4 was the reference quartile in all analyses.

Supplementary table S11: Categorical Associations between biomarkers (bio-ADM, MR-proADM, ADM-Ratio, PAM-AMA) and dementia risk after exclusion of cases occurring within the first two years of follow-up and stratification by quartiles: Univariable and multivariable-adjusted Cox regression in the BMI<30 subgroup.

|  |  | **Incident Alzheimer Dementia** | | | | **Incident Vascular Dementia** | | | |
| --- | --- | --- | --- | --- | --- | --- | --- | --- | --- |
|  | **n** | Univariable Cox regression | | Multivariable-adjusted Cox regression | | Univariable Cox regression | | Multivariable-adjusted Cox regression | |
| **Biomarker quartiles** |  | **aHR (95% CI)** | **p-value** | **aHR (95% CI)** | **p-value** | **aHR (95% CI)** | **p-value** | **aHR (95% CI)** | **p-value** |
| **PAM-AMA** | | | | | | | | | |
| p-value linear trend (LR-Chi² / LR-p value) | 0.019 (5.63 / 0.13) | | | 0.018 | | 0.72 (1.85 / 0.60) | | 0.37 | |
| Q1 (3.8 – 10.8 Units) | 901 | 1.67 (1.04 – 2.67) | 0.03 | 1.69 (1.05 – 2.71) | 0.03 | 1.03 (0.54 – 1.96) | n.s. (0.93) | 1.21 (0.63 – 2.32) | n.s. (0.57) |
| Q2 (10.8 – 12.4 Units) | 900 | 1.45 (0.90 – 2.36) | n.s. (0.13) | 1.45 (0.89 – 2.36) | n.s. (0.13) | 0.95 (0.50 – 1.83) | n.s. (0.89) | 1.14 (0.59 – 2.20) | n.s. (0.70) |
| Q3 (12.4 – 14.5 Units) | 900 | 1.14 (0.69 – 1.90) | n.s. (0.61) | 1.14 (0.69 – 1.90) | n.s. (0.60) | 0.66 (0.32 – 1.36) | n.s. (0.26) | 0.71 (0.34 – 1.46) | n.s. (0.35) |
| Q4 (14.5 – 72.1 Units) | 900 | Reference | | Reference | | Reference | | Reference | |
| **MR-proADM** | | | | | | | | | |
| p-value linear trend (LR-Chi² / LR-p value) | 0.29 (1.19 / 0.75) | | | 0.59 | | <0.0001 (16.42 / 0.001) | | 0.38 | |
| Q1 (0.121 – 0.597 nM) | 901 | 0.78 (0.48 – 1.26) | n.s. (0.31) | 1.17 (0.68 – 2.00) | n.s. (0.58) | 0.26 (0.11 – 0.60) | 0.002 | 0.72 (0.29 – 1.78) | n.s. (0.47) |
| Q2 (0.597 – 0.687 nM) | 900 | 0.82 (0.52 – 1.31) | n.s. (0.41) | 1.10 (0.67 – 1.82) | n.s. (0.70) | 0.35 (0.17 – 0.72) | 0.004 | 0.74 (0.34 – 1.62) | n.s. (0.45) |
| Q3 (0.687 – 0.811 nM) | 900 | 0.88 (0.57 – 1.37) | n.s. (0.58) | 1.09 (0.69 – 1.72) | n.s. (0.72) | 0.68 (0.39 – 1.20) | n.s. (0.18) | 1.01 (0.56 – 1.83) | n.s. (0.97) |
| Q4 (0.811 – 4.376 nM) | 900 | Reference | | Reference | | Reference | | Reference | |
| **Bio-ADM** | | | | | | | | | |
| p-value linear trend (LR-Chi² / LR-p value) | 0.06 (3.65 / 0.3) | | | 0.034 | | 0.69 (1.11 / 0.78) | | 0.09 | |
| Q1 (8 – 9.8 pg/mL) | 901 | 1.50 (0.94 – 2.40) | n.s. (0.09) | 1.62 (0.99 – 2.66) | n.s. (0.054) | 1.00 (0.54 – 1.88) | n.s. (0.99) | 1.66 (0.85 – 3.24) | n.s. (0.14) |
| Q2 (9.8 – 12.8 pg/mL) | 900 | 1.31 (0.81 – 2.11) | n.s. (0.28) | 1.34 (0.82 – 2.18) | n.s. (0.25) | 0.89 (0.47 – 1.70) | n.s. (0.73) | 1.14 (0.59 – 2.21) | n.s. (0.70) |
| Q3 (12.9 – 17.1 pg/mL) | 900 | 1.08 (0.65 – 1.77) | n.s. (0.77) | 1.07 (0.65 – 1.77) | n.s. (0.78) | 0.51 (0.24 – 1.09) | n.s. (0.08) | 0.61 (0.28 – 1.31) | n.s. (0.20) |
| Q4 (17.1 – 283 pg/mL) | 900 | Reference | | Reference | | Reference | | Reference | |
| **ADM-Ratio (x10^-^³)** | | | | | | | | | |
| p-value linear trend (LR-Chi² / LR-p value) | 0.01 (7.02 / 0.07) | | | 0.08 | | 0.02 (8.02 / 0.05) | | 0.13 | |
| Q1 (0.752 – 2.49) | 901 | 1.87 (1.16 – 3.01) | 0.01 | 1.55 (0.94 – 2.54) | n.s. (0.08) | 1.91 (1.00 – 3.66) | 0.050 | 1.45 (0.74 – 2.85) | n.s. (0.28) |
| Q2 (2.49 – 3.07) | 900 | 1.42 (0.86 – 2.35) | n.s. (0.17) | 1.29 (0.77 – 2.14) | n.s. (0.33) | 1.17 (0.57 – 2.40) | n.s. (0.66) | 1.12 (0.54 – 2.32) | n.s. (0.76) |
| Q3 (3.07 – 3.90) | 900 | 1.30 (0.77 – 2.18) | 0.32 | 1.20 (0.71 – 2.02) | n.s. (0.49) | 0.75 (0.33 – 1.68) | n.s. (0.48) | 0.70 (0.31 – 1.58) | n.s. (0.38) |
| Q4 (3.90 – 98.4) | 900 | Reference | | Reference | | Reference | | Reference | |
| **Interaction-Term ADM-Ratio x PAM-AMA (x10^-3^)** | | | | | | | | | |
| p-value linear trend (LR-Chi² / LR-p value) | <0.0001 (19.37 / <0.0001) | | | 0.001 | | 0.051 (5.17 / 0.16) | | 0.14 | |
| Q1 (10.3 – 29.7 Units) | 901 | 2.93 (1.75 – 4.90) | <0.0001 | 2.49 (1.48 – 4.21) | 0.001 | 1.68 (0.86 – 3.29) | n.s. (0.13) | 1.48 (0.75 – 2.93) | n.s. (0.26) |
| Q2 (29.7 – 38.1 Units) | 900 | 1.96 (1.14 – 3.39) | 0.02 | 1.05 (1.05 – 3.18) | 0.03 | 1.45 (0.73 – 2.90) | n.s. (0.29) | 1.41 (0.70 – 2.83) | n.s. (0.34) |
| Q3 (38.1 – 50.6 Units) | 900 | 1.70 (0.97 – 2.97) | n.s. (0.06) | 0.95 (0.95 – 2.92) | n.s. (0.07) | 0.82 (0.37 – 1.81) | n.s. (0.62) | 0.86 (0.39 – 1.89) | n.s. (0.70) |
| Q4 (50.7 – 1989 Units) | 900 | Reference | | Reference | | Reference | | Reference | |

Bio-ADM: bioactive Adrenomedullin, MR-proADM: mid-regional pro-Adrenomedullin, ADM-Ratio: molar ratio of bio-ADM/MR-proADM, PAM-AMA: peptidylglycine-alpha amidating monooxygenase amidating activity. BMI: Body-Mass Index, HR: hazard ratio, CI: confidence interval, LR-Chi²: Likelihood-Ratio Chi², n.s.: not significant. Q1-Q4: Quartiles. The HRs are shown with 95% CI, Q4 was the reference quartile in all analyses.

Supplementary table S12: Categorical Associations between biomarkers (bio-ADM, MR-proADM, ADM-Ratio, PAM-AMA) and dementia risk after exclusion of cases occurring within the first two years of follow-up and stratification by quartiles: Univariable and multivariable-adjusted Cox regression in the BMI≥30 subgroup.

|  |  | **Incident Alzheimer Dementia** | | | | **Incident Vascular Dementia** | | | |
| --- | --- | --- | --- | --- | --- | --- | --- | --- | --- |
|  | **n** | Univariable Cox regression | | Multivariable-adjusted Cox regression | | Univariable Cox regression | | Multivariable-adjusted Cox regression | |
| **Biomarker quartiles** |  | **aHR (95% CI)** | **p-value** | **aHR (95% CI)** | **p-value** | **aHR (95% CI)** | **p-value** | **aHR (95% CI)** | **p-value** |
| **PAM-AMA** | | | | | | | | | |
| p-value linear trend (LR-Chi² / LR-p value) | 0.56 (0.42 / 0.94) | | | 0.88 | | 0.27 (7.04 / 0.07) | | 0.41 | |
| Q1 (3.8 – 10.9 Units) | 236 | 0.67 (0.16 – 2.80) | n.s. (0.58) | 0.90 (0.21 – 3.86) | n.s. (0.89) | N/A | N/A | N/A | N/A |
| Q2 (10.9 – 12.5 Units) | 235 | 0.87 (0.23 – 3.23) | n.s. (0.83) | 1.01 (0.27 – 3.82) | n.s. (0.99) | 1.51 (0.52 – 9.05) | n.s. (0.65) | 2.06 (0.34 – 12.5) | n.s. (0.43) |
| Q3 (12.5 – 14.5 Units) | 235 | 1.01 (0.29 – 3.51) | n.s. (0.98) | 1.10 (0.32 – 3.85) | n.s. (0.88) | 2.50 (0.48 – 12.9) | n.s. (0.27) | 2.87 (0.55 – 15.0) | n.s. (0.21) |
| Q4 (14.5 – 29.6 Units) | 236 | Reference | | Reference | | Reference | | Reference | |
| **MR-proADM** | | | | | | | | | |
| p-value linear trend (LR-Chi² / LR-p value) | 0.02 (5.74 / 0.13) | | | 0.16 | | 0.46 (1.16 / 0.76) | | 0.81 | |
| Q1 (0.259 – 0.673 nM) | 236 | 0.25 (0.05 – 1.20) | n.s. (0.08) | 0.36 (0.06 – 1.98) | n.s. (0.24) | 0.37 (0.04 – 3.52) | n.s. (0.38) | 0.84 (0.08 – 9.44) | n.s. (0.89) |
| Q2 (0.673 – 0.782 nM) | 235 | 0.24 (0.05 – 1.18) | n.s. (0.07) | 0.37 (0.07 – 1.95) | n.s. (0.24) | 1.01 (0.21 – 5.03) | n.s. (0.99) | 2.37 (0.39 – 14.3) | n.s. (0.35) |
| Q3 (0.783 – 0.952 nM) | 235 | 0.57 (0.19 – 1.75) | n.s. (0.33) | 0.59 (0.18 – 1.90) | n.s. (0.38) | 0.95 (0.19 – 4.71) | n.s. (0.95) | 1.34 (0.26 – 6.95) | n.s. (0.73) |
| Q4 (0.953 – 3.281 nM) | 236 | Reference | | Reference | | Reference | | Reference | |
| **Bio-ADM** | | | | | | | | | |
| p-value linear trend (LR-Chi² / LR-p value) | 0.25 (7.60 / 0.055) | | | 0.50 | | 0.72 (9.18 / 0.03) | | 0.46 | |
| Q1 (8 – 14.4 pg/mL) | 236 | 0.76 (0.17 – 3.39) | n.s. (0.71) | 0.96 (0.20 – 4.51) | n.s. (0.96) | 1.33 (0.54 – 1.88) | n.s. (0.99) | 1.87 (0.43 – 8.06) | n.s. (0.40) |
| Q2 (14.4 – 18.5 pg/mL) | 235 | 0.25 (0.03 – 2.26) | n.s. (0.22) | 0.34 (0.04 – 3.15) | n.s. (0.34) | N/A | N/A | N/A | N/A |
| Q3 (18.5 – 24.5 pg/mL) | 235 | 2.17 (0.67 – 7.04) | n.s. (0.20) | 2.49 (0.74 – 8.42) | n.s. (0.14) | 0.24 (0.03 – 2.17) | n.s. (0.21) | 0.29 (0.03 – 2.66) | n.s. (0.27) |
| Q4 (24.6 – 278 pg/mL) | 236 | Reference | | Reference | | Reference | | Reference | |
| **ADM-Ratio (x10^-^³)** | | | | | | | | | |
| p-value linear trend (LR-Chi² / LR-p value) | 0.44 (3.39 / 0.34) | | | 0.49 | | 0.16 (6.86 / 0.08) | | 0.23 | |
| Q1 (1.42 – 3.06) | 236 | 1.40 (0.44 – 4.41) | n.s. (0.56) | 1.38 (0.41 – 4.58) | n.s. (0.60) | 2.05 (0.38 – 11.2) | n.s. (0.41) | 1.93 (0.33 – 11.4) | n.s. (0.47) |
| Q2 (3.06 – 3.82) | 235 | 0.64 (0.15 – 2.66) | n.s. (0.54) | 0.63 (0.15 – 2.68) | n.s. (0.53) | 2.09 (0.38 – 11.4) | n.s. (0.39) | 2.04 (0.37 – 11.4) | n.s. (0.42) |
| Q3 (3.83 – 4.78) | 235 | 0.40 (0.08 – 2.05) | n.s. (0.27) | 0.39 (0.08 – 2.04) | n.s. (0.27) | N/A | N/A | N/A | N/A |
| Q4 (4.78 – 45.1) | 236 | Reference | | Reference | | Reference | | Reference | |
| **Interaction-Term ADM-Ratio x PAM-AMA (x10^-3^)** | | | | | | | | | |
| p-value linear trend (LR-Chi² / LR-p value) | 0.99 (3.37 / 0.34) | | | 0.88 | | 0.24 (2.34 / 0.51) | | 0.28 | |
| Q1 (6.5 – 36.6 Units) | 236 | 1.06 (0.34 – 3.30) | n.s. (0.92) | 1.14 (0.36 – 3.65) | n.s. (0.83) | 2.09 (0.38 – 11.4) | n.s. (0.39) | 2.01 (0.35 – 11.6) | n.s. (0.44) |
| Q2 (36.6 – 48.1 Units) | 235 | 0.34 (0.07 – 1.68) | n.s. (0.19) | 0.40 (0.08 – 2.09) | n.s. (0.28) | 1.48 (0.25 – 8.85) | n.s. (0.67) | 2.01 (0.32 – 12.5) | n.s. (0.45) |
| Q3 (48.1 – 63.6 Units) | 235 | 0.49 (0.12 – 1.96) | n.s. (0.31) | 0.51 (0.13 – 2.04) | n.s. (0.34) | 0.49 (0.04 – 5.37) | n.s. (0.56) | 0.58 (0.05 – 6.50) | n.s. (0.66) |
| Q4 (63.6 – 700.5 Units) | 236 | Reference | | Reference | | Reference | | Reference | |

Bio-ADM: bioactive Adrenomedullin, MR-proADM: mid-regional pro-Adrenomedullin, ADM-Ratio: molar ratio of bio-ADM/MR-proADM, PAM-AMA: peptidylglycine-alpha amidating monooxygenase amidating activity. BMI: Body-Mass Index, HR: hazard ratio, CI: confidence interval, LR-Chi²: Likelihood-Ratio Chi², n.s.: not significant. Q1-Q4: Quartiles. The HRs are shown with 95% CI, Q4 was the reference quartile in all analyses.
